# Ni(I) and Ni(II) Bis(trimethylsilyl)amides Obtained in Pursuit of the Elusive Structure of Ni{N(SiMe_3_)_2_}_2_

**DOI:** 10.1021/acs.inorgchem.3c04483

**Published:** 2024-05-06

**Authors:** Connor P. McLoughlin, Anthony J. Witt, Philip P. Power

**Affiliations:** Department of Chemistry, University of California, Davis, California 95616, United States

## Abstract

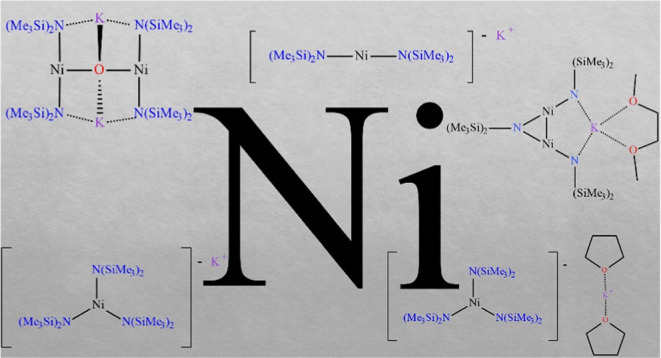

Salt metathesis routes to five new –N(SiMe_3_)_2_ nickel derivatives were studied to illuminate their mode of formation, structures, and spectroscopy. The reaction between NiI_2_ and K{N(SiMe_3_)_2_} afforded the Ni(II) and Ni(I) complexes [K][Ni{N(SiMe_3_)_2_}_3_] (**1**) and [K][Ni{N(SiMe_3_)_2_}_2_] (**2**). Dissolving **1** in tetrahydrofuran (THF) gave the Ni(II) species [K(THF)_2_][Ni{N(SiMe_3_)_2_}_3_] (**3**). The Ni(I) salt [K(DME)][Ni_2_{N(SiMe_3_)_2_}_3_] (**4**) was obtained by using NiCl_2_(DME) (DME = 1,2-dimethoxyethane) as the nickel source rather than NiI_2_. The isolation of the Ni(I) complexes **2** and **4** highlights the tendency for K{N(SiMe_3_)_2_} to function as a reducing agent. Introduction of adventitious O_2_ to solutions of [K][Ni{N(SiMe_3_)_2_}_2_] (**2**) gave the nickel inverse crown ether (ICE) species [K_2_][O(Ni{N(SiMe_3_)_2_}_2_)_2_] (**5**). Complex **5** is the first ICE complex of nickel and is one of four known ICE complexes for the 3d metals. The experimental results indicate that the reduced Ni(I) bis(trimethylsilyl)amides are relatively easily generated, whereas Ni(III) derivatives that might be expected from a disproportionation of a Ni(II) derivative are apparently not yet isolable by the above routes. Overall, the new species crystallize readily from the reaction mixtures, but under ambient conditions, they begin to decompose as solids within ca. 24 h, which hinders their characterization.

## Abstract 2

The reaction between Ni(II) halides and K{N(SiMe_3_)_2_}, in lieu of Na{N(SiMe_3_)_2_}, afforded two Ni(II) and two Ni(I) complexes. Introduction of adventitious O_2_ to solutions of the Ni(I) species [K][Ni{N(SiMe_3_)_2_}_2_] gave the Ni(II) inverse crown ether (ICE) [K_2_][O(Ni{N(SiMe_3_)_2_}_2_)_2_].

## Introduction

In 2015, it was shown that the earlier report of the synthesis of Ni{N(SiMe_3_)_2_}_2_ in tetrahydrofuran (THF) solvent by Bürger and Wannagat^1^ actually described its THF complex Ni{N(SiMe_3_)_2_}_2_(THF) instead of THF free, uncomplexed Ni{N(SiMe_3_)_2_}_2_.^2^ This finding was consistent with the formation of the corresponding Mn(II), Fe(II), and Co(II) THF complexes in THF solvent.^3^ While several homoleptic Ni(II) amides are known,^4−12^ the structure of the silylamide species Ni{N(SiMe_3_)_2_}_2_ has remained elusive, and its usefulness as a synthon is limited by its apparent instability.^2^ The number of currently known neutral Lewis base complexes of Ni{N(SiMe_3_)_2_}_2_ is limited,^13^^1^ and the isolation of such complexes is strongly dependent upon the solvent employed. The other types of Ni{N(SiMe_3_)_2_}_2_ derivatives are either Ni(II) or Ni(I) nickelate salts.^2^ For example, Ni{N(SiMe_3_)_2_}_2_(dmap)_2_ (dmap = 4-dimethylaminopyridine) and Ni{N(SiMe_3_)_2_}_2_ (2,2′-bipy) (2,2′-bipy = 2,2′-bipyridine) were isolated by Werncke and co-workers via the addition of dmap or 2,2′-bipy in toluene to the nickelate [Li(THF)_′4.5–5.5′_][Ni{N(SiMe_3_)_2_}_3_].^14^ Reduction of [Li(THF)_′4.5–5.5′_][Ni{N(SiMe_3_)_2_}_3_]^14^ with KC_8_ (KC_8_ = potassium graphite) in Et_2_O and 18-crown-6 (18-crown-6 = 1,4,7,10,13,16-hexaoxacyclooctadecane) gave the Ni(I) complex [K(18-crown-6)][Ni{N(SiMe_3_)_2_}_2_] while reduction in toluene gave trace amounts of the Ni–Ni bonded species [K(toluene)][Ni_2_{N(SiMe_3_)_2_}_3_].^14^ Additionally, attempts to isolate [Na][Ni{N(SiMe_3_)_2_}_3_] were unsuccessful in the absence of donor ligands.^15^ It was suggested that the nature of the donor ligand is critical for the initial formation of the nickelate.^15^ The dependence of the formation of these Ni(II) silylamides on solvent effects and the use of chelating agents to form separated ion pairs prompted us to investigate further the isolation of donor ligand-free derivatives of Ni{N(SiMe_3_)_2_}_2_. Herein, we report the syntheses of four new Ni(II) and Ni(I) complexes of the –N(SiMe_3_)_2_ ligand that do not require the use of complexing agents and whose isolations are determined solely by the reaction conditions. Complex **5** was obtained from the reaction of [K][Ni{N(SiMe_3_)_2_}_2_] (**2**) with molecular O_2_.

## Experimental Section

### General Considerations

All manipulations were carried out under anaerobic and anhydrous conditions by using standard Schlenk techniques or in a Vacuum Atmospheres OMNI-Lab drybox under an atmosphere of dry argon or nitrogen. Solvents were dried by the method of Grubbs and co-workers,^16^ stored over potassium or sodium, and then degassed by the freeze–pump–thaw method. All physical measurements were made under strictly anaerobic and anhydrous conditions. Melting points of samples in flame-sealed capillaries were determined using a Meltemp II apparatus equipped with a partial immersion thermometer and a device limit of 250 °C. Infrared (IR) spectra were recorded as Nujol mulls between CsI plates on a PerkinElmer 1430 spectrometer. Ultraviolet–visible (UV–vis) spectra were recorded as dilute toluene solutions in 3.5 mL quartz cuvettes using an Olis 17 modernized Cary 14 UV–Vis–near-IR spectrophotometer. NiI_2_ and K{N(SiMe_3_)_2_} were obtained from commercial sources and used as received. The purity of the K{N(SiMe_3_)_2_}, which was found to resonate at ca. 0.14 ppm in C_6_D_6_, was confirmed by ^1^H NMR spectroscopy. NiCl_2_(DME) was prepared according to a literature procedure.^17^ NMR spectra were recorded on a Bruker 400 MHz AVANCE III HD Nanobay spectrometer, and the ^1^H NMR spectra were referenced to the residual solvent signals in deuterated benzene or deuterated toluene. Magnetic susceptibility data were collected at room temperature by the Evans method^18^ and were corrected using the appropriate diamagnetic constants.^19^ Powder X-ray diffraction (PXRD) patterns for **1** and **2** were collected on a Bruker D8 Advance diffractometer using Cu Kα radiation operated at 40 kV and 25 mA at room temperature.

### Synthesis

#### [K][Ni(N(SiMe_3_)_2_)_3_] (**1**)

0.295 g of NiI_2_ (0.944 mmol) and 0.529 g of K{N(SiMe_3_)_2_} (2.65 mmol) were combined in a 100 mL Schlenk flask with cooling to ca. 0 °C. A ca. 35 mL solution of chilled (ca. 0 °C) Et_2_O was added via a cannula to the reaction flask to give a gray suspension. This suspension was stirred for 15 min at ca. 0 °C and was then warmed to room temperature and stirred for a further ca. 18 h. The solvent was removed under reduced pressure (ca. 0.01 Torr) to leave a dark green solid. The residue was washed with ca. 10 mL of hexane and dried under reduced pressure for 1 h. Extraction of the dark green solids with ca. 40 mL of hexane produced a red solution that was filtered via a filter-tipped cannula. The filtrate was concentrated to ca. 25 mL, whereupon microcrystalline material precipitated onto the walls of the flask. This was redissolved at room temperature, and the solution was stored overnight at ca. −18 °C to give yellow needles of **1** that were suitable for X-ray crystallographic studies. Solutions of **1** begin to decompose above ca. −10 °C. In the absence of solvent, the crystals slowly decompose over ca. 12 h. Yield: 0.086 g (16%) mp 85–87 °C (dec). μ_eff_: 2.37 μ_B_. ^1^H NMR (400 MHz, C_7_D_8_, 25 °C) 10.74 ppm ^1^H NMR (400 MHz, C_6_D_6_, 25 °C) 10.75 ppm. UV–vis λ/nm (ε/M^–1^ cm^–1^): 225 nm (6800), 408 nm (3000), 489 nm (2300). IR (Nujol; ṽ/cm^–1^) 2900 (s), 2720 (m), 1450 (s), 1375 (s), 1255 (s), 1240 (s), 1210 (s), 970 (s), 830 (s), 780 (s), 755 (s), 740 (s), 705 (s), 670 (s), 655 (s), 620 (m), 610 (m), 395 (w), 370 (m).

#### [K][Ni(N(SiMe_3_)_2_)_2_] (**2**)

0.299 g of NiI_2_ (0.956 mmol) and 0.572 g of K{N(SiMe_3_)_2_} (2.868 mmol) were combined in a 100 mL Schlenk flask with cooling to ca. 0 °C. A ca. 35 mL solution of chilled (ca. 0 °C) Et_2_O was added via a cannula to the reaction flask to give a gray suspension. This suspension was stirred for 15 min at ca. 0 °C and warmed to room temperature. After ca. 1 h, the suspension became green and was stirred for a further 18 h at room temperature. The solvent was removed under reduced pressure (ca. 0.01 Torr) to leave a dark green solid. The residue was washed with ca. 10 mL of hexane and dried under reduced pressure for 1 h. Extraction of the dark green solid with ca. 40 mL of hexane produced a red solution that was filtered via a filter-tipped cannula. This red solution was stored overnight in a ca. 8 °C fridge to give teal blocks of **2** that were suitable for X-ray crystallographic studies. Redissolving the crystals of **2** at room temperature in hexane, benzene, or toluene yields the slow decomposition of **2**. Crystalline **2** is stable for ca. 24 h. at room temperature. mp 183–185 °C (dec.) 0.026 g (7%). μ_eff_: 1.7 μ_B_^1^H NMR (400 MHz, C_6_D_6_, 25 °C, ppm) 0.77, 0.04, −0.36. UV–vis λ/nm (ε/M^–1^ cm^–1^): 225 nm (1400). IR (Nujol; ṽ/cm^–1^) 2900 (s), 1470 (s), 1380 (s), 1250 (s), 1185 (m), 1050 (s), 985 (s), 940 (s), 835 (s), 785 (s), 755 (s), 715 (m), 670 (s), 620 (m), 380 (m).

#### [K(THF)_2_][Ni(N(SiMe_3_)_2_)_3_] (**3**)

A solution of **1** was synthesized from 0.303 g of NiI_2_ (0.970 mmol) and 0.581 g of K{N(SiMe_3_)_2_} (2.91 mmol). Then, 0.039 g of crystalline **1** was dissolved in ca. 50 mL of hexane, and a 1:2 stoichiometric (Ni/THF) quantity of THF (0.01 mL) was added to the solution via a syringe and stirred for ca. 2 h. The solvent was concentrated to ca. 15 mL, which afforded a deposit of yellow microcrystalline material on the walls of the Schlenk flask. The solution was stored overnight in a ca. −35 °C freezer to give yellow, feather-like plates of **3** that were suitable for X-ray crystallography. Crystalline **3** is stable at room temperature for ca. 48 h, but ^1^H NMR spectroscopy shows partial decomposition at ca. 25 °C. 0.024 g (48%) mp 51–53 °C (dec.). μ_eff_: 2.24 μ_B_. ^1^H NMR (400 MHz, C_6_D_6_, 25 °C, ppm) 10.70. UV–vis λ/nm (ε/M^–1^ cm^–1^): 223 (3400), 402 (650), 487 (660). IR (Nujol; ṽ/cm^–1^) 2900 (s), 2840 (s), 1455 (s), 1370 (s), 1255 (s), 1170 (m), 1085 (s), 1010 (s), 975 (m), 925 (m), 795 (s), 715 (m), 610 (w), 365 (w).

#### [K(DME)][Ni_2_(N(SiMe_3_)_2_)_3_] (**4**)

0.203 g (0.924 mmol) of NiCl_2_(DME)^17^ and 0.515 g (2.58 mmol) of K{N(SiMe_3_)_2_} were combined in a 100 mL Schlenk flask with cooling to ca. 0 °C. Chilled (ca. 0 °C) Et_2_O (ca. 35 mL) was added via a cannula to the reaction flask to give a pale green suspension. The suspension was stirred for 15 min at ca. 0 °C and warmed to room temperature, whereupon stirring was continued for ca. 12 h. The solvent was removed under reduced pressure (ca. 0.01 Torr) to leave a green solid. This residue was washed with ca. 10 mL hexane and dried under reduced pressure for 1 h. Extraction of the dark green solids with ca. 40 mL of hexane produced a red solution that was filtered via a filter-tipped cannula. Storage of the red solution in a ca. 8 °C fridge for 2 days, followed by storage in a ca. −18 °C freezer for 3 weeks, gave bright red crystals of **4** that were suitable for X-ray crystallography. Crystalline **4** is stable in solution at −18 °C, but as a room temperature solid, it begins to decompose to black after ca. 24 h. Yield 0.033 g (10%, calculated from NiCl_2_(DME)) of **4**, mp 111–113 °C. ^1^H NMR (400 MHz, C_6_D_6_, 25 °C, ppm) 3.03, 2.87, 1.39, 0.98, 0.92. μ_eff_: 1.20 μ_B_. UV–vis λ/nm (ε/M^–1^ cm^–1^): 226 (1400). IR (Nujol; ṽ/cm^–1^) 2900 (s), 1450 (s), 1370 (s), 1250 (s), 1085 (s), 1000 (s, broad) 975 (s), 880 (s), 830 (s, broad), 750 (m), 720 (m), 700 (m), 670 (m), 610 (w), 440 (w), 360 (w).

#### [K_2_][O(Ni{N(SiMe_3_)_2_}_2_)_2_] (**5**)

The synthesis of **2** (see above) was repeated with 0.304 g (0.971 mmol) of NiI_2_ and 0.503 g (2.52 mmol) of K{N(SiMe_3_)_2_}. The hexane solution of in situ synthesized **2** was placed in a ca. −18 °C freezer for 3 weeks, after which the silicone grease seal had become eroded. Colorless crystals that were suitable for X-ray crystallography were recovered from this solution to yield 0.018 g (4%, calculated from NiI_2_) of **5**, mp 70–71 °C. μ_eff_: 1.46 μ_B_. ^1^H NMR (400 MHz, C_6_D_6_, 25 °C, ppm) 0.38. UV–vis λ/nm (ε/M^–1^ cm^–1^): 225 nm (5800). IR (Nujol; ṽ/cm^–1^) 2920 (s), 2840 (s), 1460 (s), 1375 (s), 1260 (s), 1180 (m), 1015 (s), 930 (s), 840 (s), 800 (s), 385 (w).

### X-ray Crystallographic Studies

Crystals of **1**–**5** suitable for X-ray crystallographic studies were obtained from saturated hexane solutions at various temperatures (see above). The crystals were removed from the Schlenk tubes and immediately covered with a layer of hydrocarbon oil. Suitable crystals were selected, mounted on a nylon cryoloop, and then placed in the cold nitrogen stream of the diffractometer. Data for **3** and **5** were collected at 100(2) K, and data for **1**, **2**, and **4** were collected at 190(2) K. Data for **3** were collected with Cu Kα_1_ radiation (λ = 1.5418 A), while data for **1**, **2**, and **4**–**5** were collected with Mo Kα_1_ radiation (λ = 0.71073 Å) using a Bruker D8 Venture dual source diffractometer in conjunction with a CCD detector. The collected reflections were corrected for Lorentz and polarization effects and for absorption by using Blessing’s method as incorporated into the program SADABS.^20,21^ The structures were solved by direct methods and refined with the SHELXTL (2012, version 6.1) or SHELXTL (2013) software packages.^22^ Refinement was done by full-matrix least-squares procedures, with all carbon-bound hydrogen atoms included in calculated positions and treated as riding atoms. The thermal ellipsoid plots were drawn using OLEX2 software.^23^

## Results and Discussion

### Synthesis and Structures

**Figure 1:**
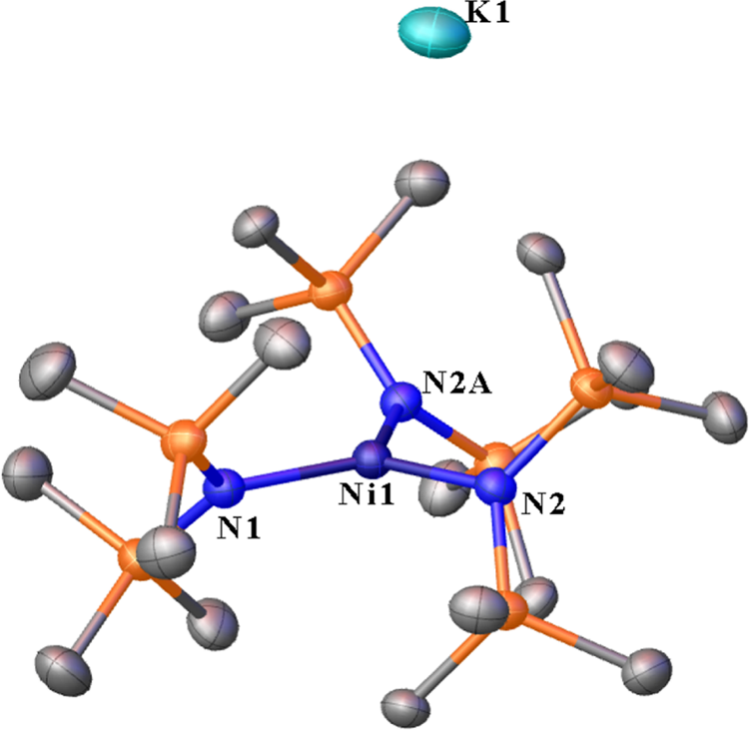
A 50% thermal ellipsoid plot of [K][Ni{N(SiMe_3_)_2_}_3_] (**1**). Hydrogen atoms are not shown for clarity. Selected distances (Å) and angles (°): Ni1–N1 1.934(2), Ni1–N2 1.9299(16), Ni1–N2A 1.9299(16), N1–Ni1–N2 121.13(5), N1–Ni1–N2A 121.12(5), and N2–Ni1–N2A 117.75(9). ∑_angles_ Ni1 = 360.00(11).

The complex [K][Ni{N(SiMe_3_)_2_}_3_] (**1**) is readily isolated (see above) as bright yellow needles upon cooling a hexane solution of **1** to ca. −18 °C (Figure 1). These yellow needles can be removed under a counter-flow of the working gas (Ar or N_2_) at room temperature for X-ray crystallographic analysis without noticeable decomposition. However, the stability of **1** in the Paratone oil used in the protection of the crystals is limited to ca. 30 min at room temperature. Nonetheless, this stability is remarkable considering that the analogous species [Na][Ni{N(SiMe_3_)_2_}_3_] cannot be isolated without Lewis base complexation of the sodium cation.^15^ Complex **1** is the first isolable Ni(II) bis(trimethylsilyl)amide salt free of complexation by neutral donor solvent molecules bound to the Ni atom or donor-sequestered alkali metal cations. The isolation of **1** as a solid updates previous reports of donor-free Ni(II) and Ni(I) complexes, which were reported to exist as oils at room temperature or to decompose readily in the absence of solvent or under reduced pressure.^2,14,15^ Hexane solutions of [K][Ni{N(SiMe_3_)_2_}_3_] (**1**) retain a red hue for more than 1 week at temperatures less than ca. −10 °C. Benzene solutions of **1** decompose after ca. 12 h at room temperature, and toluene solutions of **1** decompose after ca. 12 h at or above 0 °C. In further contrast to previous reports, the synthesis of other 3-coordinate Ni(II) nickelate salts required the use of THF^14^ or pmdeta^15^ as a solvent or the use of other base-stabilized Ni(II) species, such as Ni{N(SiMe_3_)_2_}_2_(dmap)_2_, as starting materials.^14^

**Figure 2:**
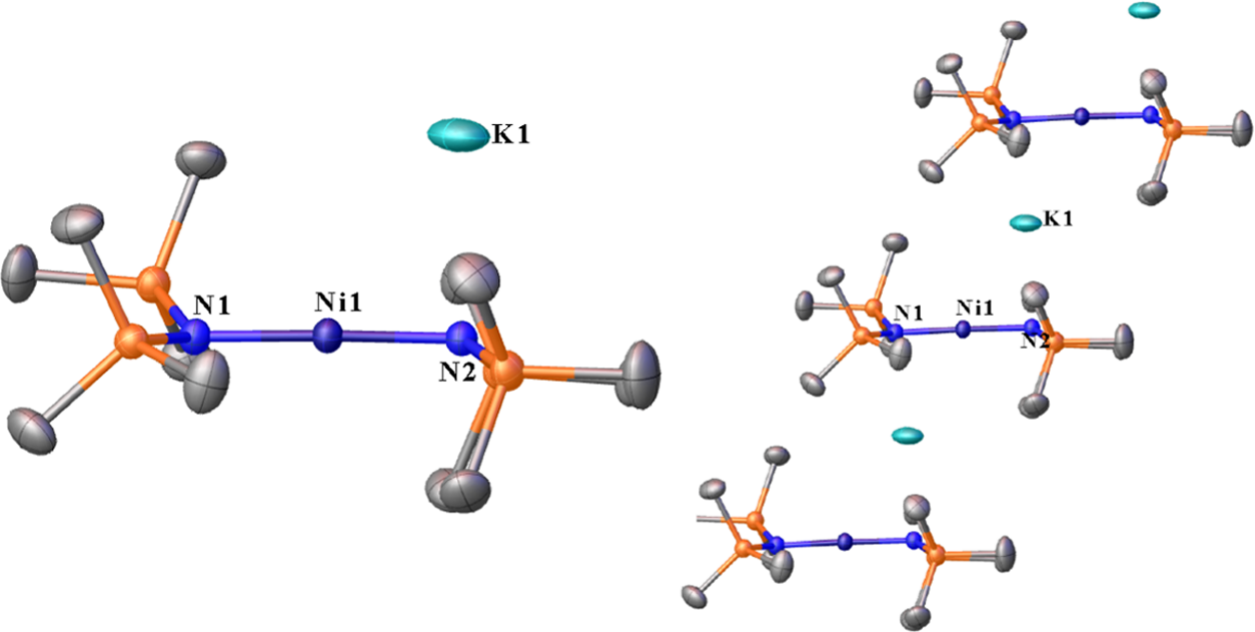
Left: 50% thermal ellipsoid plot of [K][Ni{N(SiMe_3_)_2_}_2_] (**2**). Right: Side view of a 50% thermal ellipsoid plot of [K][Ni{N(SiMe_3_)_2_}_2_] (**2**) showing the connectivity between each molecule in their discrete asymmetric units. Hydrogen atoms are not shown for clarity. Selected distances (Å) and angles (°): Ni1–N1 1.8559(9), Ni1–N2 1.8711(9), and K1···N2 2.8370(9). N1–Ni1–N2 176.81(4).

After a solution of **1** was stored for ca. 1 week in a benzene-*d*_6_ solution, teal crystals were recovered from the J. Young NMR tube. Analysis of the crystals by X-ray crystallography gave a structure of the Ni(I) species [K][Ni{N(SiMe_3_)_2_}_2_] (**2**) (Figure 2). The complex [K][Ni{N(SiMe_3_)_2_}_2_] (**2**) is a Ni(I) nickelate that is not stabilized by the coordination of donor solvents or sequestered cations. The initial isolation of **2** from the NMR tube gave crystals in a quantity that only permitted an X-ray crystallographic characterization. Investigations of the intentional formation of the Ni(I) complex [K][Ni{N(SiMe_3_)_2_}_2_] (**2**) in higher yield revealed that at temperatures below ca. −10 °C, a 3:1 ligand to metal ratio gives complex **1** with no observable formation of teal-colored crystals. However, repeating this reaction with the same 3:1 ligand to metal salt ratio, followed by storage at ca. 8 °C gave pale, teal-colored blocks of [K][Ni{N(SiMe_3_)_2_}_2_] (**2**) as the only crystalline product (Scheme 2). Storing solutions of **1** at room temperature also gave crystalline **2**, consistent with its initial isolation but in lower crystalline yield.

Thus, the formation of **2** from solutions of **1** is likely the result of a one-electron reduction of an in situ generated Ni{N(SiMe_3_)_2_}_2_ by the excess equivalent of K{N(SiMe_3_)_2_} rather than by the degradation of Ni{N(SiMe_3_)_2_}_2_ or other intermediates, with the rate of reduction being temperature-dependent. Potassium salts of organic nucleophiles have been shown to reduce similar species,^24^ but the Ni(I) tetramer^2^ [Ni{N(SiMe_3_)_2_}]_4_ was not observed as a side product in this case. The reported investigations of other potential mechanisms of formation revealed that the use of the powerful reducing agent KC_8_ with [Li(THF)_′4.5–5.5′_][Ni{N(SiMe_3_)_2_}_3_]^14^ in toluene gave a Ni(I) complex [K(toluene)][Ni_2_{N(SiMe_3_)_2_}_3_].^14^ It was suggested that this complex could be viewed as an intermediate in the decomposition pathway to the Ni(I) tetramer [Ni{N(SiMe_3_)_2_}]_4_.^2,14^ While there are some structural similarities between **2** and [K(toluene)][Ni_2_{N(SiMe_3_)_2_}_3_],^14^ such as the sub-180° N–Ni–N angles and the sub-1.9 Å Ni–N distances, the solution behavior of interest cannot be compared at present due to the difficulty in isolating [K(toluene)][Ni_2_{N(SiMe_3_)_2_}_3_] in quantities suitable for thorough spectroscopic characterization.^14^

**Figure 3:**
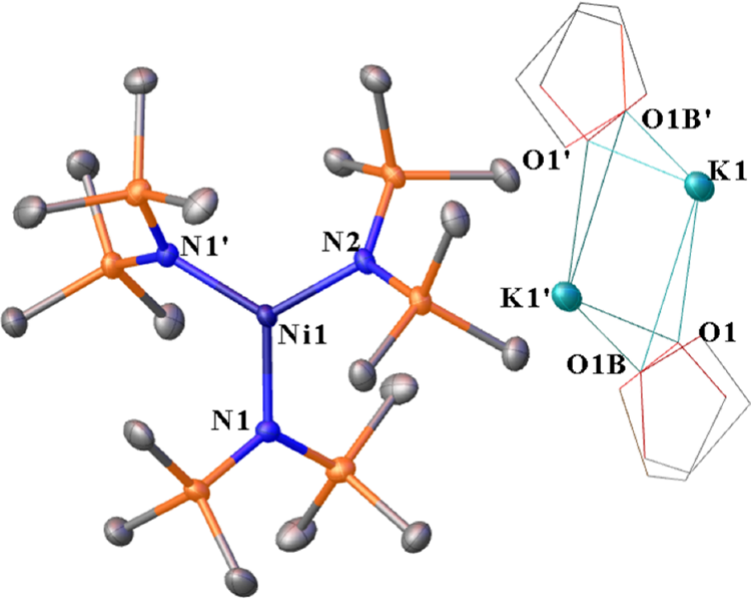
Thermal ellipsoid plot (50%) of [K(THF)_2_][Ni{N(SiMe_3_)_2_}_3_] (**3**) with solvent molecules (THF) shown in their disordered positions as wireframe structures. Hydrogen atoms are not shown for clarity; K1 (light blue) is disordered over two positions (K1 and K1′), each with 50% occupancy. Selected distances (Å) and angles (°): Ni1–N1 1.9339(16), Ni1–N2 1.927(2), Ni1–N1′ 1.9339(16), Ni1···K1 6.4053(11), Ni1···K1′ 7.5809(10), N1–Ni1–N2 119.96(5), N1–Ni1–N1′ 120.07(10), N2–Ni1–N1′ 119.96(5), and ∑Ni1 359.99(12). O1–K1′ 2.595(8), O1–K1 3.279(8), O1B–K1 2.609(9), and O1B–K1′ 3.302(8).

**Scheme 1:**
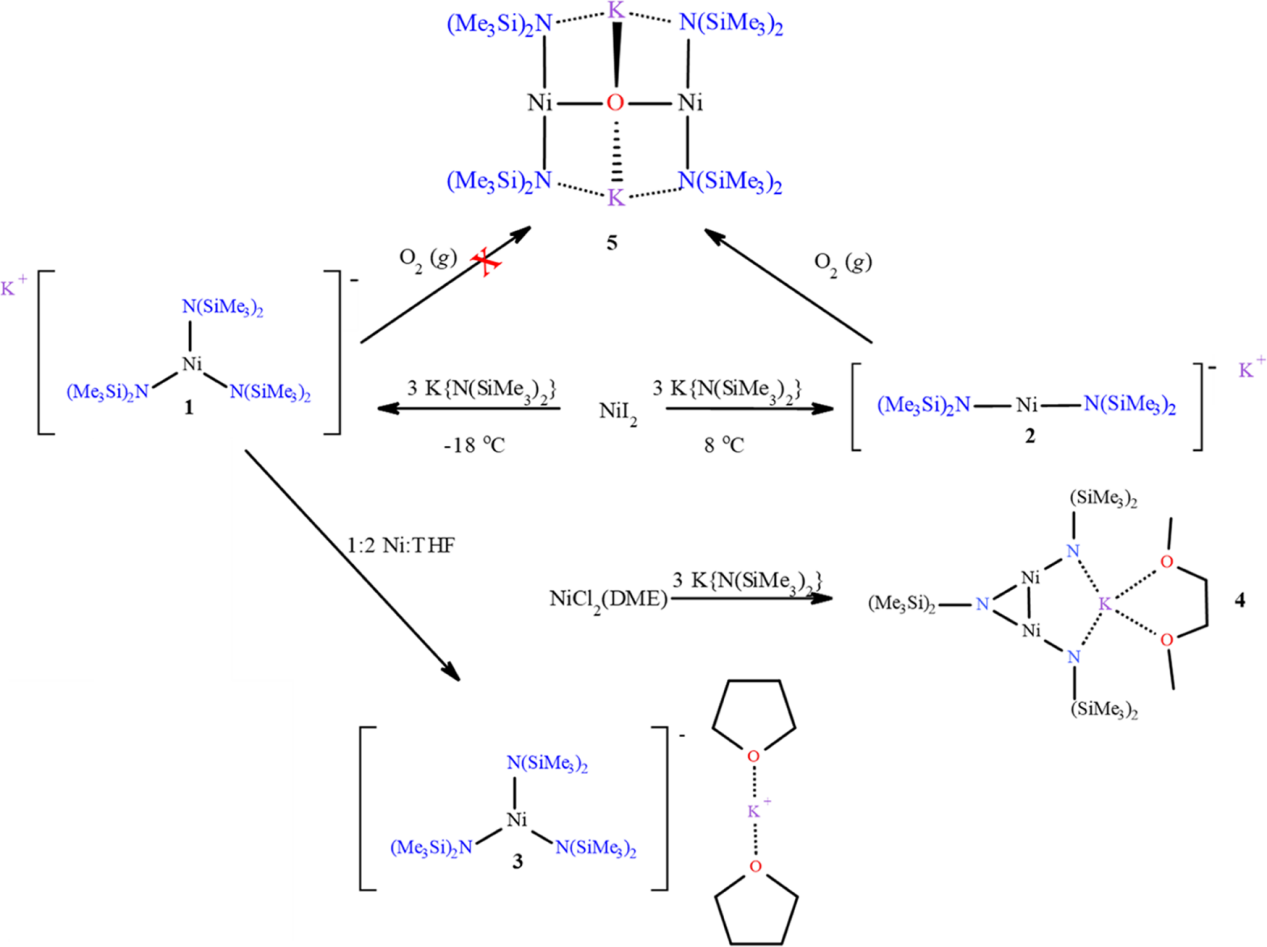
Reaction Summary for the Synthesis of Complexes **1**–**5**

To determine if coordinating solvents prevent the formation of a 3-coordinate Ni(II) nickelate species, a stoichiometric amount of THF (Scheme 1) was added to a hexane solution of **1**. Storage of this solution at ca. −35 °C gave yellow, feather-like crystals. Analysis of these crystals via X-ray crystallography revealed them to be [K(THF)_2_][Ni{N(SiMe_3_)_2_}_3_] (**3**). The structure of the [Ni{N(SiMe_3_)_2_}_3_]^−^ anion in **3** (Figure 3) is nearly identical to that of **1**, but the countercation features THF coordination to the K^+^ ion. This result is surprising, as we expected that the coordination of THF to the Ni^2+^ ion would occur rapidly in solution and give the known compound Ni{N(SiMe_3_)_2_}_2_(THF).^2^ This further emphasizes the significance of the transfer agent cation and the solvent type in the isolation of new complexes.

**Figure 4:**
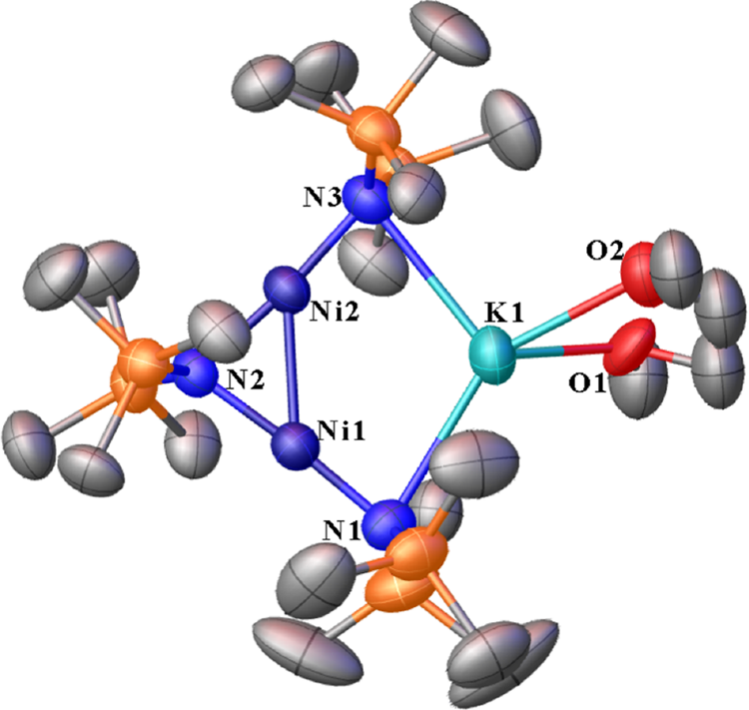
Thermal ellipsoid plot (50%) of [K(DME)][Ni_2_{N(SiMe_3_)_2_}_3_] (**4**). Hydrogen atoms and minor occupancy positions of the –SiMe_3_ groups and DME atoms are not shown for clarity. Selected distances (Å) and angles (°): Ni1···K1 3.3051(7), Ni2···K1 3.2759(8). Ni1–Ni2 2.4109(5), Ni1–N1 1.8660(19), Ni1–N2 1.8921(17), Ni2–N2 1.9005(15), Ni2–N3 1.8607(17), N1–K1 3.052(2), N3–K1 3.0539(19), N1–Ni1–N2 175.83(8), N2–Ni2–N3 174.00(8), N3–Ni2–Ni1 135.62(6), N1–Ni1–Ni2 133.48(6), Ni1–N2–Ni2 78.94(6), N2–Ni2–Ni1 50.38(5), N2–Ni1–Ni2 50.68(5), and N1–K1–N3 110.77(5).

While a previous attempt to synthesize Ni{N(SiMe_3_)_2_}_2_ using NiCl_2_ and K{N(SiMe_3_)_2_} as the transfer agent in a 3:1 ligand to metal ratio gave intractable brown solids,^14^ addition of K{N(SiMe_3_)_2_} to NiCl_2_(DME) in a 3:1 ligand to metal ratio followed by extraction of the resultant residue with hexanes gave red crystals of [K(DME)][Ni_2_{N(SiMe_3_)_2_}_3_] (**4**, Figure 4) in low yield. No other crystalline compounds were isolated from this reaction. The structure of **4** is analogous to that of [K(toluene)][Ni_2_{N(SiMe_3_)_2_}_3_]^14^ reported by Werncke and co-workers. Whereas, the complex [K(toluene)]{[Ni_2_{N(SiMe_3_)_2_}_3_]} was isolated from the KC_8_ reduction of [Li(THF)_′4.5–5.5′_][Ni{N(SiMe_3_)_2_}_3_] in toluene.^14^ The propensity for K{N(SiMe_3_)_2_} to function as a reducing agent is further demonstrated in the isolation of **4**, given that its toluene congener is only observed upon reduction with the potent reducing agent KC_8_.^14^

**Figure 5:**
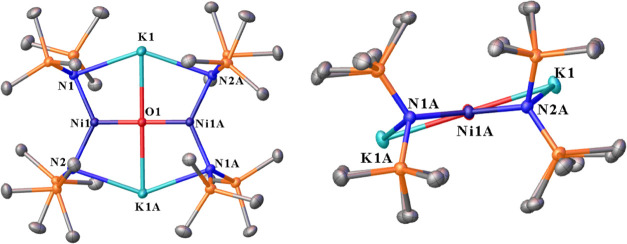
Thermal ellipsoid plot (50%) of [K_2_][O(Ni{N(SiMe_3_)_2_}_2_)_2_] (**5**) with hydrogen atoms not shown for clarity. Selected distances (Å) and angles (°): Ni1–O1 1.80152(19), Ni1–N2 1.9330(11), Ni1–N1 1.9362(11), K1–O1 2.6873(3), K1–N2A 2.7923(11), K1–N1 2.8381(11), Ni1···K1 3.2675(4), Ni1–K1A···3.2028(4), O1–Ni1–N2 115.90(3), O1–Ni1–N1 113.92(3), N2–Ni1–N1 130.18(5), Ni1–O1–Ni1A 180.00, Ni1A–O1-K1 88.761(8), Ni1–O1–K1 91.238(9), Ni1A–O1-K1A 91.239(8), Ni1–O1–K1A 88.762(9), and K1–O1–K1A 180.00.

During attempts to repeat the synthesis of [K][Ni{N(SiMe_3_)_2_}_2_] (**2**), a flask containing 3 equiv of K{N(SiMe_3_)_2_} and 1 equiv of NiI_2_ was stored in a ca. −18 °C freezer for 3 weeks. After this period, it was removed from the freezer, and it was observed that the silicone grease of the glass stopper had become “streaky” and partially eroded. Removal of the supernatant liquid via a cannula revealed a cluster of colorless crystals that had been deposited on the wall of the flask that were suitable for characterization via X-ray crystallography. These crystals allowed the structure of the new Ni(II) complex [K_2_][O(Ni{N(SiMe_3_)_2_}_2_)_2_] (**5**) to be determined. The formation of complex **5** (Figure 5) is likely due to adventitious amounts of oxygen or moisture that arose during storage of the Schlenk flask in the freezer, producing an imperfect silicone grease seal at the glass stopper. In the “inverse crown” ether (ICE) structure, the metals in the “crown” act as Lewis acids for the O^2–^ anion.^25,26^ To date, the principal isolation routes of all ICE complexes of the first-row transition metals have been reported to involve the introduction of “adventitious” amounts of oxygen, similar to the isolation route for complex **5**.^27,28^ Furthermore, the syntheses designed to isolate pure ICE complexes of the 3d metals give impure products for Mn(II) and Co(II), but pure ICE complexes of Zn(II) can be isolated.^27−29^ For Ni(II), storage of three separate hexane solutions of [K][Ni{N(SiMe_3_)_2_}_2_] (**2**) in a −18 °C freezer with an imperfect silicone grease seal reliably gave colorless crystals of **5**.

**Scheme 2:**
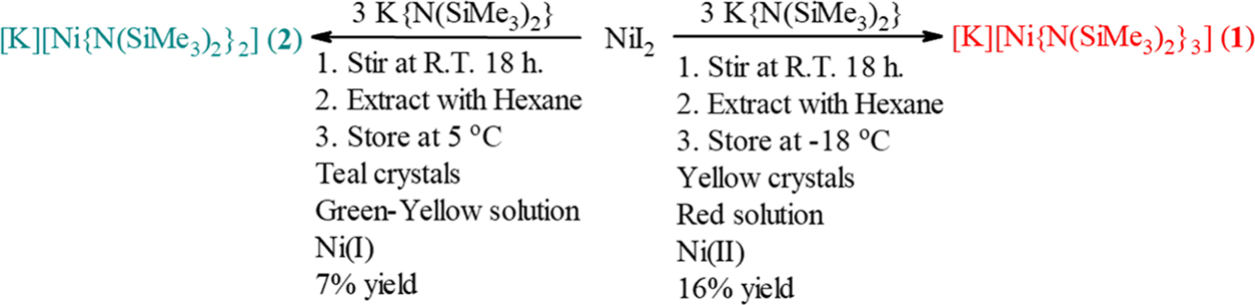
Synthesis of Compounds **1** and **2**

The structure of **5** resembles those of the rare 3d metal inverse crown ether (ICE) complexes of Mn(II),^27^ Co(II),^28^ and Zn(II) reported by Mulvey and co-workers.^29^ The bis(trimethylsilyl)amido ICE complexes are limited to a Mn(II) complex with Na^+^ cations,^27^ a Co(II) complex with Na^+^ cations,^28^ and two Zn(II) complexes.^29^ A further Mn(II) ICE complex was isolated using 2,2,6,6-tetramethylpiperidide as the amido anion.^27^ A few other ICE complexes exist for s- and p-block^30−34^ metals and one f-block ytterbium ICE complex.^35^ The complex **5**, however, is the first example for nickel (Scheme 2).

### Structural Analysis

In complex **1**, the K^+^ ion is associated with the anion via C–H···K^+^ contacts. The sum of the interligand bond angles at the Ni(II) atom in [K][Ni{N(SiMe_3_)_2_}_3_] (**1**) are within a standard deviation of 360° and are indicative of a trigonal planar geometry. The Ni–N distances (av. 1.931(2) Å) in **1** are similar to those reported for neutral and anionic Ni(II) complexes.^2,14,15^ However, the distances are near the upper end of the range of Ni–N(SiMe_3_)_2_ distances of ca. 1.86–1.95 Å (Table 1). The K^+^ ions are “solvated” by 12 adjacent (K···H < 3.1 Å) hydrogens from 6 of the ligand methyl groups. Two of the three bis(trimethylsilyl)amido ligands at each Ni(II) atom participate in this solvation of K^+^ ions by the discrete [Ni{N(SiMe_3_)_2_}_3_]^−^ units.

**Table 1:** Selected Distances (Å) and Angles (°) in Ni(I) and Ni(II) Bis(trimethylsilyl)amides

compound	Ni–N	Ni-donor	N–Ni–N	coord. no. and structure type	∑_angles_ Ni
[K][Ni{N(SiMe_3_)_2_}_3_] (**1**)	1.931(2)^a^	N/A	120(2)^a^	3, ion pair	359.999(8)
[K][Ni{N(SiMe_3_)_2_}_2_] (**2**)	1.86(1)^a^	N/A	176.81(6)	2, ion pair	176.81(6)
[K(THF)_2_][Ni{N(SiMe_3_)_2_}_3_] (**3**)	1.932(4)	N/A	120.00(6)	3, ion pair	359.99(12)
[K(DME)][Ni_2_{N(SiMe_3_)_2_}_3_] (**4**)	1.88(2)^a^	K-donor	175(1)	3, monomer	360.00(12)
[K_2_][O(Ni{N(SiMe_3_)_2_}_2_)_2_] (**5**)*	1.935(2)^a^	N/A	130.18(5)	3, monomer	360.00(6)
Ni{N(SiMe_3_)_2_}_2_(THF)^2^	1.861(3)^a^	2.0143(2)	140.664(5)	3, monomer	359.86(27)
Ni{N(SiMe_3_)_2_}_2_(py)_2_^2^	1.942(4)^a^	1.9310(6)^a^	179.2607(3)	4, monomer	358.3599(4)
[Ni{N(SiMe_3_)_2_}]_4_^2^	1.916(3)^a^	N/A	168.85(7)^a^	2, tetramer	168.85(7)^a^
	1.9197(14)	1.96(6)^a^	N/A	4, monomer	360.07
[Na(pmdeta)_2_][Ni{N(SiMe_3_)_2_}_3_]^15^	1.929(2)^a^	Na-donor	120.0(7)^a^	3, ion pair	359.9(2)
[Li(dmap)_4_][Ni{N(SiMe_3_)_2_}_3_]^14^	1.930(2)^a^	Li-donor	120(2)^a^	3, ion pair	360.00(8)
Ni{N(SiMe_3_)_2_}_2_(dmap)_2_^14^	1.949(1)^a^	1.917(1)^a^	179.08(6)	4, monomer	356.64(8)
[Ni{N(SiMe_3_)_2_}(bipy)]^14^	1.898(3)	1.947(4)^a^	N/A	3, monomer	360.01(31)
Ni{N(SiMe_3_)_2_}_2_(bipy)^14^	1.860(1)	2.030(1)	136.05(6)	4, monomer	415.05(10)
[K(18-crown-6)][Ni{N(SiMe_3_)_2_}_2_]^14^	1.866(1)	K-donor	180	2, ion pair	180
[K(toluene)][Ni_2_{N(SiMe_3_)_2_}_3_]^14^	1.881(16)	K-donor	174.74(5)	3, monomer	359.91(7)

a Average value *triclinic data. dmap = 4-dimethylaminopyridine; bipy = 2,2′-bipyridine; pmdeta = *N*,*N*,*N*′,*N*″,*N*″-pentamethyldiethylenetriamine.

Only one 2-coordinate Ni(I) amido anion stabilized exclusively by bis(trimethylsilyl)amido ligands has been isolated to date, and this features either 18-crown-6 or [2.2.2]cryptand sequestered K^+^ countercations for a [Ni{N(SiMe_3_)_2_}_2_]^−^ anion. Notably, the previously reported Ni(I) complex K{(18-crown-6)}[Ni{N(SiMe_3_)_2_}_2_]^14^ (or K{[2.2.2]cryptand}[Ni{N(SiMe_3_)_2_}_2_]^14^) has the only strictly linear coordinated Ni(I) anion with a 180° N–Ni–N angle (Table 1). The N–Ni–N angle between the bis(trimethylsilyl)amido ligands in [K][Ni{N(SiMe_3_)_2_}_2_] (**2**) is 176.81(6)^o^, and its deviation from linearity, although slight, is the largest of the related 2-coordinate complexes (Table 1). The Ni–N distances of 1.8559(9) and 1.8711(9) Å differ slightly. The solvation of the K^+^ ion in **2** by the ligands and the K^+^···N distance of ca. 2.86 Å implicates the coordination of the K^+^ ion by the lone-pair electrons of the nitrogen atom (N2, Figure 2) as the cause of the deviation from linearity. This close contact between the nitrogen atom and K cation causes a slight pyramidalization of the geometry at the N atoms, which is not observed in other 2-coordinate Ni(I) species. Unexpectedly, the Ni–N distances in **2** are shorter than those reported for other Ni(I) amido species, and just two Ni(II) species have shorter Ni–N distances (cf. Table 1).

[K(THF)_2_][Ni{N(SiMe_3_)_2_}_3_] (**3**) is the only nickel bis(trimethylsilyl)amido complex featuring a donor solvent (in this case, THF) coordinating to an atom other than nickel. The K^+^ ions are disordered over two 50% occupancy positions. The solvent molecules reflect the split occupancy of the K^+^ ions in the lattice and are also disordered over 51% and 49% (Figure 3) occupancy positions. The sum of the interligand angles at the Ni(II) atom of 359.99(12)° confirms a trigonal planar geometry. This sum of angles is identical to that observed in **1** and is within a standard deviation to those observed in Ni(II) nickelate anions featuring donor-sequestered cations (Table 1). Two of the three Ni–N distances are identical at 1.9339(16) Å, while one is slightly shorter at 1.927(2) Å to give an average Ni–N distance of 1.932(4) Å. These Ni–N distances are within the expected range^2,14,15^ of ca. 1.86–1.95 Å (Table 1) for Ni(II) species and are identical within standard deviations to those observed in complex **1**.

For [K(DME)][Ni_2_{N(SiMe_3_)_2_}_3_] (**4**), the Ni1–Ni2 distance of 2.4108(6) Å is shorter than that observed in [K(toluene)][Ni{N(SiMe_3_)_2_}_3_]^14^ by ca. 0.03 Å. This Ni1–Ni2 distance is also shorter than those observed in the Ni(I) tetramer [Ni{N(SiMe_3_)_2_}]_4_^2^ by ca. 0.02 Å. The Ni–N distances are comparable to those in [K(toluene)][Ni{N(SiMe_3_)_2_}_3_]^14^ but shorter than those in [Ni{N(SiMe_3_)_2_}]_4_.^2^ Given that the structure of [K(toluene)][Ni{N(SiMe_3_)_2_}_3_]^14^ and [K(DME)][Ni_2_{N(SiMe_3_)_2_}_3_] (**4**) can be viewed as intermediate structures between neutral Ni{N(SiMe_3_)_2_}_2_ and [Ni{N(SiMe_3_)_2_}]_4_,^2,14^ the shorter Ni–N distances are expected due to a decrease in steric repulsion of the –SiMe_3_ groups compared to those in the Ni(I) tetramer [Ni{N(SiMe_3_)_2_}]_4_.^2^ The effective magnetic moment of 1.20 μ_B_ for [K(DME)][Ni_2_{N(SiMe_3_)_2_}_3_] (**4**) indicates a strong antiferromagnetic coupling between the two Ni(I) ions, since the spin-only value for two distinct, noninteracting Ni(I) nuclei is 2.45 μ_B_.^36^ Crystallographic data for **4** were collected at 190(2)K, and at this temperature, **4** crystallizes in the orthorhombic space group *Pbca*. Slowly cooling the crystal on the goniometer to 100(2)K results in a temperature-dependent phase transition from orthorhombic *Pbca* to monoclinic *P*2_1_/*c*. The β angle shifts from 90° at 190(2) K to 90.285(4)° at 100(2) K. A temperature-dependent phase transition was not reported for [K(toluene)][Ni{N(SiMe_3_)_2_}_3_],^14^ and further investigation is required to determine if this observation is unique to complex **4**.

The complex [K_2_][O(Ni{N(SiMe_3_)_2_}_2_)_2_] (**5**) features a planar coordinated μ_4_-O atom that occupies a center of symmetry and affords a half-molecule per unit cell. Examination of the crystals under a microscope reveals two distinct morphologies for crystalline **5**. The first form involves colorless rectangular plates in which **5** crystallizes in the triclinic space group *P*1̅ (Figure 5). The second involves rectangular blocks that crystallize in the monoclinic space group *P*2_1_/*n*. In the triclinic crystal system, the average Ni–N distance of 1.935(2) Å is comparable to those in the Ni(II) complexes **1** and **3** within standard deviations (Table 1). The sum of the angles in **5** for the Ni and O atoms is 360.00(6)°. The K^+^ ions and the nitrogen atoms of the bis(trimethylsilyl)amido ligands in **5** are not coplanar. A planar configuration is likely unfavorable due to electrostatic and steric repulsion of the bis(trimethylsilyl)amido ligands. The average Ni···K geometrical distance for **5** in the space group P1̅ is 3.24(5) Å, and this distance lies between the geometrical distances observed in the Ni(I) complexes **2** and **4**.

### Spectroscopy

The solution-phase ^1^H NMR spectrum of **1** is of interest, since the chemical shifts of the resonances observed (Table 2) for **1** are remarkably similar to those given in the 2015 report for the synthesis of Ni{N(SiMe_3_)_2_}_2_.^2^ The ^1^H NMR signal in C_7_D_8_ assigned to Ni{N(SiMe_3_)_2_}_2_ appeared at 10.7 ppm,^2^ while the chemical shift for [K][Ni{N(SiMe_3_)_2_}_3_] (**1**) in the same solvent resonated at 10.74 ppm. A signal at 0.09 ppm, which was assigned to HN(SiMe_3_)_2_, is also observed for **1**, but there is no signal in the spectrum that indicated the formation of the Ni(I) decomposition product [Ni{N(SiMe_3_)_2_}]_4_, whose ^1^H NMR signal appears at 0.03 ppm. It was suggested^15^ that the donor ligands (pmdeta, dmap, (18-crown-6), [2.2.2]cryptand) were crucial for the formation of the reported nickelates^14,15^ and that their solution-phase equilibria with Ni{N(SiMe_3_)_2_}_2_^15^ can be tuned by the presence of these donor ligands, depending on the solvents and chelating agents used.

**Table 2:** Solution-Phase ^1^H NMR Chemical Shifts (ppm) at Room Temperature for **1**–**5** and ^1^H NMR SiMe_3_ Resonances for Known Ni(I) and Ni(II) Bis(trimethylsilyl)amides

compound	solvent	^1^H NMR chemical shift (ppm)
[K][Ni{N(SiMe_3_)_2_}_3_] (**1**)^a^	C_7_D_8_	10.74
	C_6_D_6_	10.74
[K][Ni{N(SiMe_3_)_2_}_2_] (**2**)^a^	C_6_D_6_	0.77, 0.04, −0.36
[K(THF)_2_][Ni({N(SiMe_3_)_2_}_3_)] (**3**)^a^	C_6_D_6_	10.70
[K(DME)][Ni_2_{N(SiMe_3_)_2_}_3_] (**4**)^a^	C_6_D_6_	3.03, 2.87, 1.39, 0.98, 0.92
[K_2_][O(Ni{N(SiMe_3_)_2_}_2_)_2_] (**5**)^a^	C_6_D_6_	0.38
Ni{N(SiMe_3_)_2_}_2_^2^	C_7_D_8_	10.70
Ni{N(SiMe_3_)_2_}_2_(THF)^2^	C_7_D_8_	9.79
Ni{N(SiMe_3_)_2_}_2_(py)_2_^2^	N/A	N/A
[Ni{N(SiMe_3_)_2_}]_4_^2^	C_6_D_6_	0.3
	N/A	N/A
[Na(pmdeta)_2_][Ni{N(SiMe_3_)_2_}_3_]^15^	THF-*d*_8_	1.17
[Li(dmap)_4_][Ni{N(SiMe_3_)_2_}_3_]^14^	THF-*d*_8_	1.18
Ni{N(SiMe_3_)_2_}_2_(dmap)_2_^14^	C_7_D_8_	4.24
[Ni{N(SiMe_3_)_2_}(bipy)]^14^	N/A	N/A
Ni{N(SiMe_3_)_2_}_2_(bipy)^14^	THF-*d*_8_	9.74
	C_7_D_8_	10.62
[K(18-crown-6)][Ni{N(SiMe_3_)_2_}_2_]^14^	THF-*d*_8_	0.25

a This work.

The solid-state structure of [K][Ni{N(SiMe_3_)_2_}_3_] (**1**) is unlikely to be completely maintained in solution, as indicated by the large change in color from yellow to red upon its dissolution in hexane. Additionally, the similarity of the chemical shift of the ^1^H NMR resonance at 10.74 ppm to that of Ni{N(SiMe_3_)_2_}_2_ and the presence of an HN(SiMe_3_)_2_ signal at 0.09 ppm suggest that partial dissolution has occurred with concomitant formation of Ni{N(SiMe_3_)_2_}_2_. However, no transformation to the Ni(I) species [Ni{N(SiMe_3_)_2_}]_4_ was observed. This is inconsistent with the conclusions in previous reports.^14,15^ It also demonstrates that the formation of a nickelate species is not necessarily dependent upon the presence of donor solvents,^14,15^ since none is needed to initially form or stabilize [K][Ni{N(SiMe_3_)_2_}_3_] (**1**). No signal was observed in the solution-phase ^1^H NMR spectrum of the species [Ni{N(SiMe_3_)_2_}]_4_, and no black crystals were recovered from the dark, decomposed solutions of [K][Ni{N(SiMe_3_)_2_}_3_] (**1**).

The ground-state term symbol for a d^8^ Ni(II) ion is ^3^F_4_.^37^ In the D_3h_ point group, the free ion term symbol is further split into E″, A_1_′, and E′ levels, in order of increasing energy.^37^ As such, a minimum of three absorptions are expected to be observed in the electronic spectrum. Accordingly, three maxima appear in the UV–vis spectrum of **1** at 225 nm (6800 M^–1^ cm^–1^), 408 nm (3000 M^–1^ cm^–1^), and 489 nm (2300 M^–1^ cm^–1^). The related Ni(II) species [K(THF)_2_][Ni{N(SiMe_3_)_2_}_3_] (**3**) displays very similar absorption maxima at 223 nm (3400 M^–1^ cm^–1^), 402 nm (650 M^–1^ cm^–1^), and 487 nm (660 M^–1^ cm^–1^). However, the extinction coefficients for **3** are significantly lower than those in **1** for all of the observable transitions. The ^1^H NMR spectrum of **3** reflects this significantly different solution-phase behavior compared to **1**, as the ^1^H NMR signal for **3** is shifted somewhat to 10.70 ppm (Table 2). Notably, this is essentially the same as the ^1^H NMR resonance reported for neutral Ni{N(SiMe_3_)_2_}_2_.^2^ The differences in the electronic spectra and ^1^H NMR spectra of **1** and **3**, despite their common anionic [Ni{N(SiMe_3_)_2_}_3_]^−^ moieties, is likely indicative of the nearly complete dissociation in hydrocarbon solution of [K(THF)_2_][Ni{N(SiMe_3_)_2_}_3_] (**3**) to Ni{N(SiMe_3_)_2_}_2_ and probably K(THF)_2_{N(SiMe_3_)_2_}. Unfortunately, the extinction coefficients in the electronic spectrum of Ni{N(SiMe_3_)_2_}_2_ were not reported, and further study will be needed to determine the extent to which complexes **1** and **3** dissociate in solution to form Ni{N(SiMe_3_)_2_}_2_. These results indicate that the reactions between the Ni(II) halides and alkali metal bis(trimethylsilyl)amides are a complex process that can result in several different products. The type of product obtained depends mainly on the Ni(II) halide used, the countercation of the bis(trimethylsilyl)amide ligand, and the solvent used. In this respect, the nickel system differs drastically from the corresponding Mn(II), Fe(II), and Co(II) systems.

## Conclusions

Five new nickel derivatives of –N(SiMe_3_)_2_ were synthesized via a salt metathesis of NiI_2_ or NiCl_2_(DME) and K{N(SiMe_3_)_2_} in varying stoichiometric ratios. Although all the new complexes were characterized by SC-XRD, their solution phase ^1^H NMR spectra are complicated by their tendency to decompose to give additional signals. [K][Ni{N(SiMe_3_)_2_}_3_] (**1**) and [K][Ni{N(SiMe_3_)_2_}_2_] (**2**) are the first Lewis base free ionic bis(trimethylsilyl)amido nickel complexes. Similarly, the complex [K(THF)_2_][Ni{N(SiMe_3_)_2_}_3_] (**3**) is the first to feature THF coordinating to the K^+^ ion rather than the Ni(II) atom. The formation of [K][Ni{N(SiMe_3_)_2_}_2_] (**2**) and [K(DME)][Ni_2_{N(SiMe_3_)_2_}_3_] (**4**) via a K{N(SiMe_3_)_2_} reduction emphasizes the importance of the –N(SiMe_3_)_2_ group countercation used as the transfer agent in these reactions. The propensity for Ni{N(SiMe_3_)_2_}_2_ derivatives to undergo reactions with small molecules like O_2_ is realized in the formation of the complex [K_2_][O(Ni{N(SiMe_3_)_2_}_2_)_2_] (**5**). Complex **5** is one of just 4 characterized ICE complexes for the 3d metals.

## Supporting Information Available

Spectroscopic (^1^H NMR, IR, and UV–vis) data of **1**–**5** and crystallographic tables for **1**–**5** (PDF)

## Footnotes

1 Although less common than the +2 oxidation state, Ni(I) complexes are now numerous.

2 For example, [Li(dmap)_4_][Ni(II){N(SiMe_3_)_2_}_3_]^14^ and [Na(pmdeta)_2_][Ni(II){N(SiMe_3_)_2_}_3_].^15^
