# Supplementary material for: Ni(I) and Ni(II) Bis(trimethylsilyl)amides Obtained in Pursuit of the Elusive Structure of Ni{N(SiMe3)2}2
Source: Inorg Chem. 2024 May 6;63(20):9031–9. doi: 10.1021/acs.inorgchem.3c04483 (PMC11110003; doi:10.1021/acs.inorgchem.3c04483)
Supplement: Supplementary file 1 — ic3c04483_si_001.pdf [file ic3c04483_si_001.pdf]

Supporting Information for

Ni(I) and Ni(II) Bis(trimethylsilyl)amides Obtained in Pursuit of the Elusive Structure of $Ni\{N(SiMe_3)_2\}_2$

Connor P. McLoughlin[a†], *Anthony J. Witt*[a†], *and Philip P. Power*[a]*

[a]Department of Chemistry, University of California, Davis, California 95616, United States.

[†]These authors contributed equally

Philip P. Power: pppower@ucdavis.edu

Supporting Information

Table of Contents:

1. Characterization data for complex **1**……………………………………………………...S3

Figure S1. ^{1}H NMR (C_6D_6) spectrum of **1** ……………………….……………………...S3

Figure S2. ^{1}H NMR (C_7D_8) spectrum of **1** ……………………….…….………………...S3

Figure S3. UV-vis spectrum (0.461 mM) of **1**...……………………………………………S4

Figure S4. Infrared spectrum of **1** …………………………………………………………….S4

2. Characterization data for complex **2**…………………………………………………………S5

Figure S5. ^{1}H NMR (C_6D_6) spectrum of **2** ……………………….……………………...S5

Figure S6. UV-vis spectrum (0.597 mM) of **2**...……………………………………………S5

Figure S7. Infrared spectrum of **2** …………………………………………………………….S6

3. Characterization data for complex **3** …………………………………………………...S6

Figure S8. ^{1}H NMR spectrum of **3**...……………………………………………………...S6

Figure S9. UV-vis (0.645 mM) spectrum of **3**………………………………………..…….S7

Figure S10. Infrared spectrum of **3**...……………………..……………………………....S7

4. Characterization data for complex **4** …………………………………………………...S8

Figure S11. ^{1}H NMR spectrum of **4**………………………………………………………...S8

Figure S12. UV-vis spectrum (298 μM) of **4**………………………………………………….S8

Figure S13. Infrared spectrum of **4**……………………………………………………………….S9

5. Characterization data for complex **5**……………..……………………………………………..S9

Figure S14. ^{1}H NMR spectrum of **5**………………………………………………………...S9

Figure S15. UV-vis spectrum (234 μM) of **5**...........…………………………………………S10

Figure S16. Infrared spectrum of **5**……………………………………………………………...S10

6. X-Ray Crystallographic data and structural parameters for **1-5**……………………………..S10

Table S1. Crystal data and structure refinement for **1-3**…………………………………….…….S10

Table S2. Crystal data and structure refinement for **4-5**…………………………………….…….S12

7. Photos of crystalline samples……………………………………………………………….S12

Figure S17. $[K][Ni(N(SiMe_3)_2)_3]$ (**1**)…………………………………………………………S13

Figure S18. $[K][Ni(N(SiMe_3)_2)_2]$ (**2**)…………………………………………………………S13

Figure S19. $[K(THF)_2][Ni\{N(SiMe_3)_2\}_3]$ (**3**)……………………………………………...S14

Figure S20. $[K(DME)][Ni_2\{N(SiMe_3)_2\}_3]$ (**4**)……………………………………………..S14

Figure S21. $[K_2][O(Ni\{N(SiMe_3)_2\}_2)_2]$ (**5**)……………………….……………………………….S15

8. PXRD patterns of **1** and **2**……………………………….……………………………S15

Figure S22. PXRD pattern of **1**…………………………………………………………………S15

Figure S23. PXRD pattern of **2**…………………………………………………………………S15

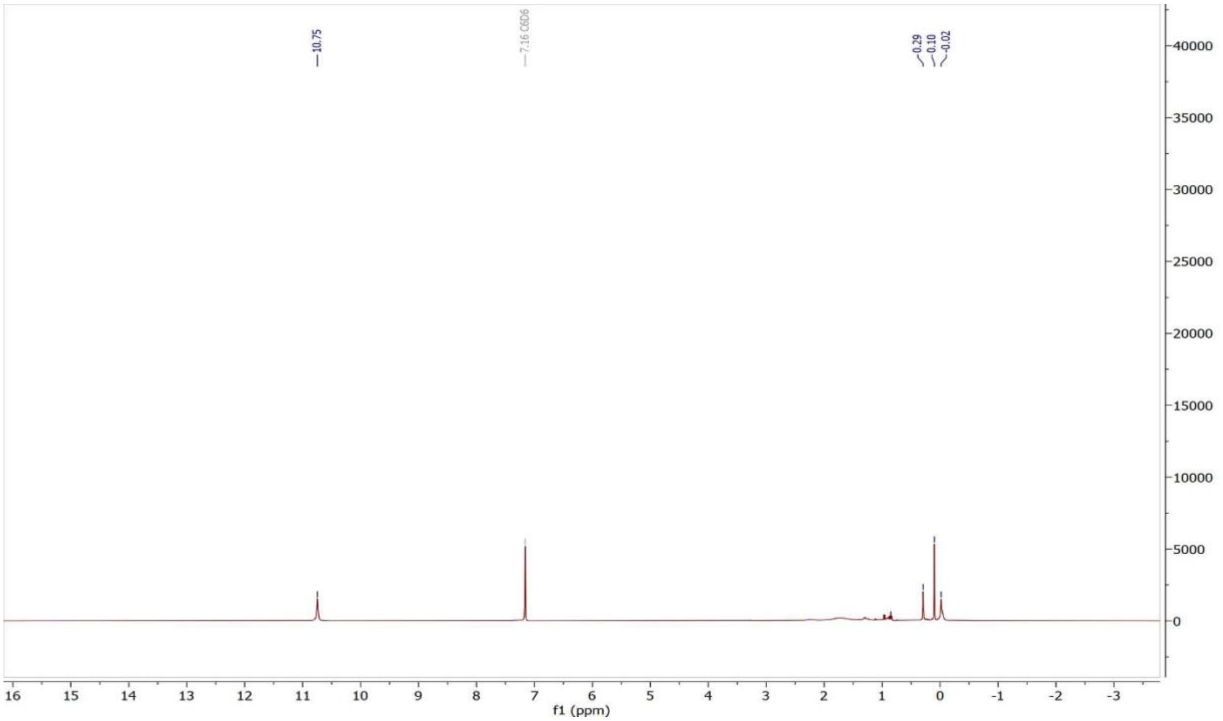

Figure S1. ^{1}H NMR (400 MHz, C_6D_6, 25 ^oC of [K][Ni(N(SiMe$_3$)$_2$)$_3$] (**1**). Silicone grease and free HMDS indicated at 0.29 and 0.10 ppm, respectively.

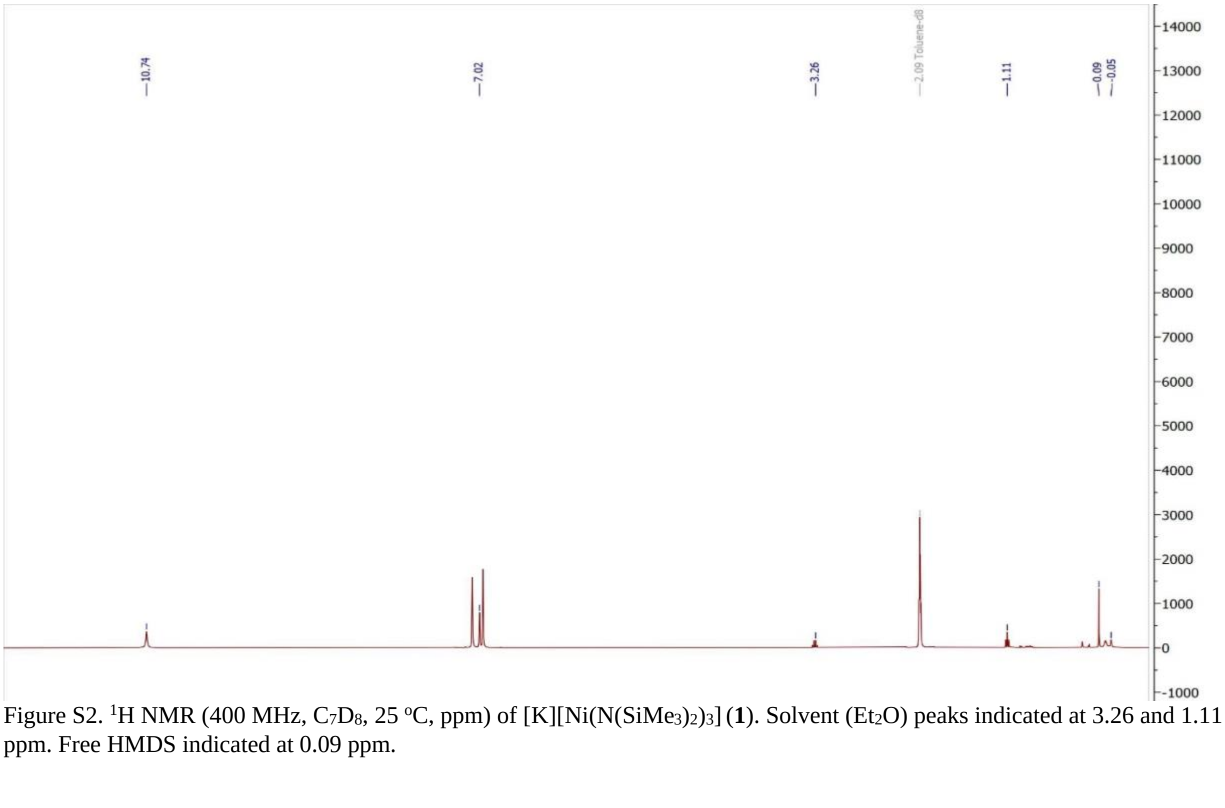

Figure S2. ^{1}H NMR (400 MHz, C_7D_8, 25 ^oC, ppm) of [K][Ni(N(SiMe$_3$)$_2$)$_3$] (**1**). Solvent (Et_2O) peaks indicated at 3.26 and 1.11 ppm. Free HMDS indicated at 0.09 ppm.

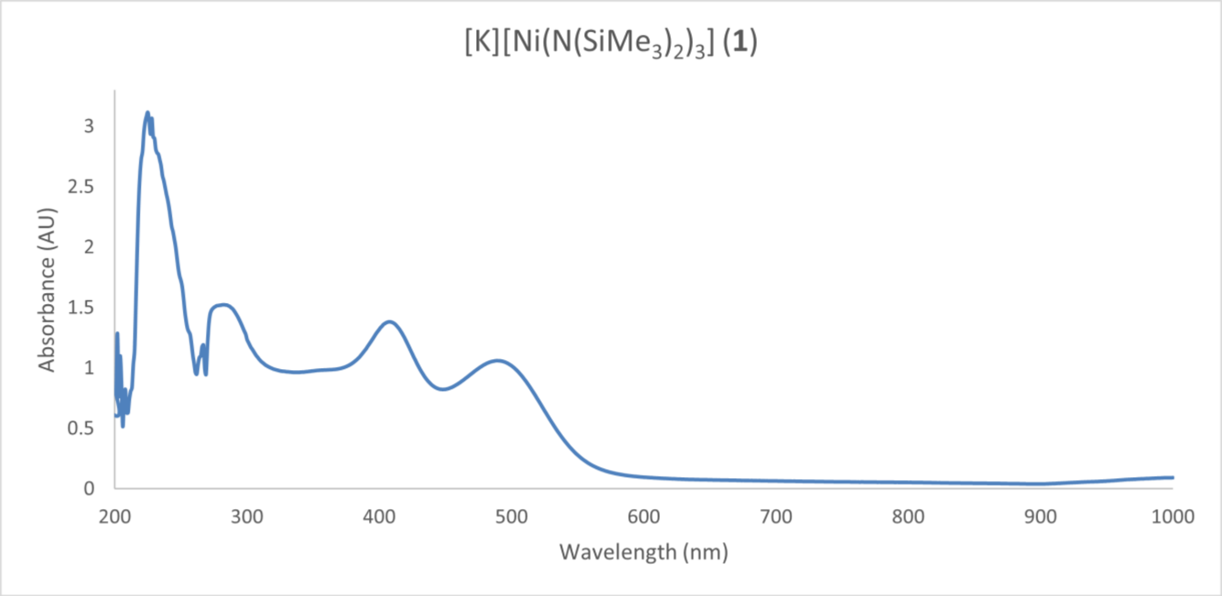

Figure S3. Concentrated (0.461 mM) UV-vis spectrum of [K][Ni(N(SiMe$_3$)$_2$)$_3$] (**1**) in hexane.

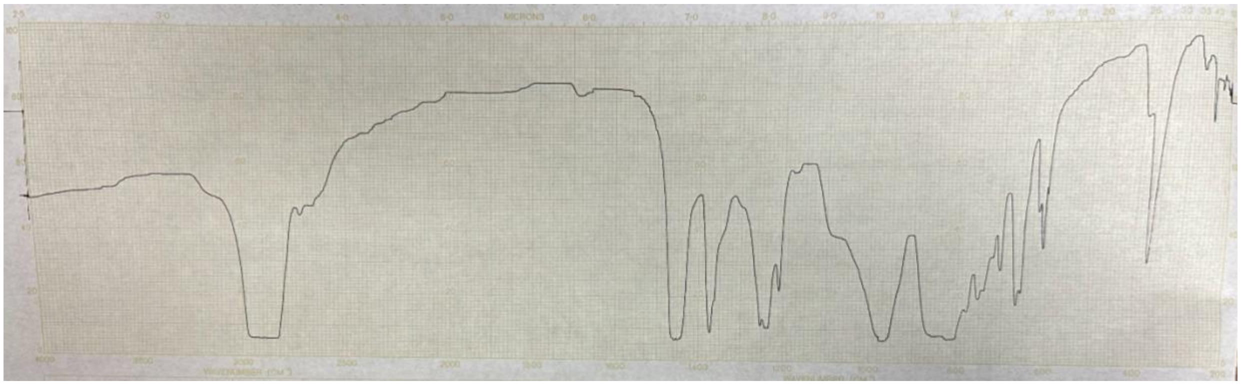

Figure S4. IR spectrum (Nujol) of [K][Ni(N(SiMe$_3$)$_2$)$_3$] (**1**).

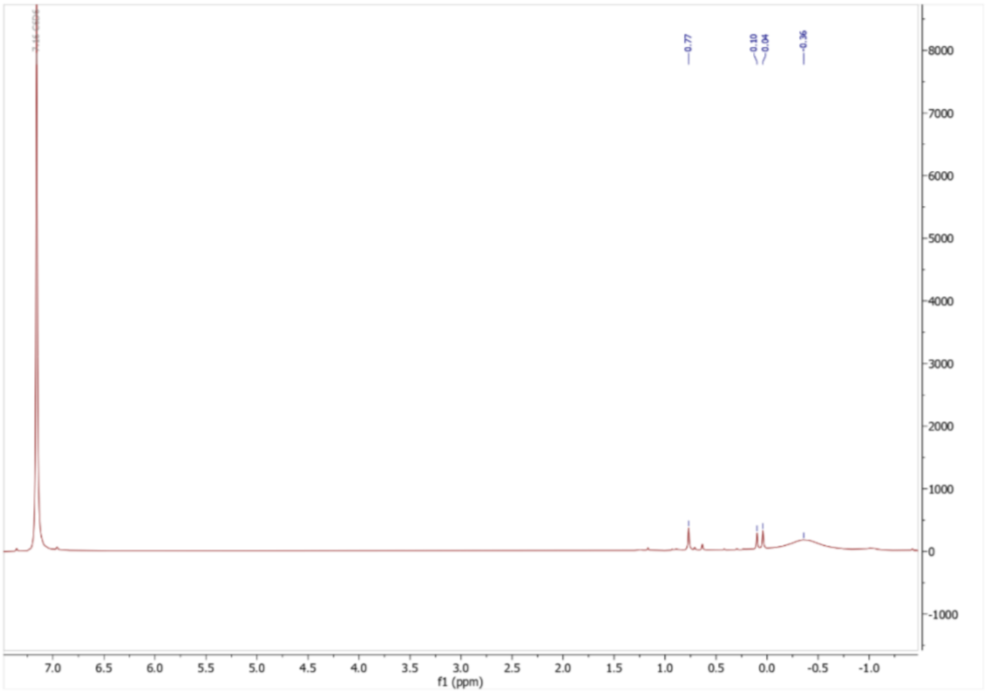

Figure S5. ^{1}H NMR (400 MHz, C_6D_6, 25 °C) spectrum of $[K][Ni(N(SiMe_3)_2)_2]$ (**2**). Free HMDS indicated at 0.10 ppm.

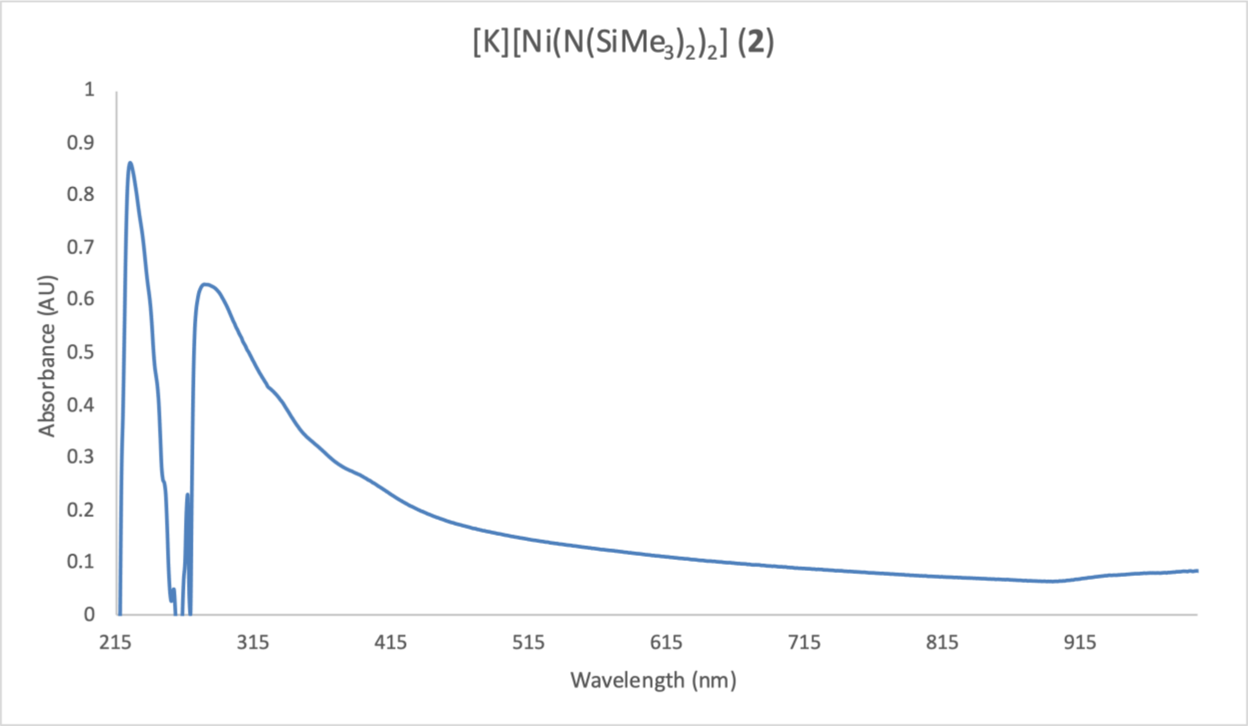

Figure S6. Concentrated (0.597 mM) UV-vis spectrum of $[K][Ni(N(SiMe_3)_2)_2]$ (**2**) in hexane.

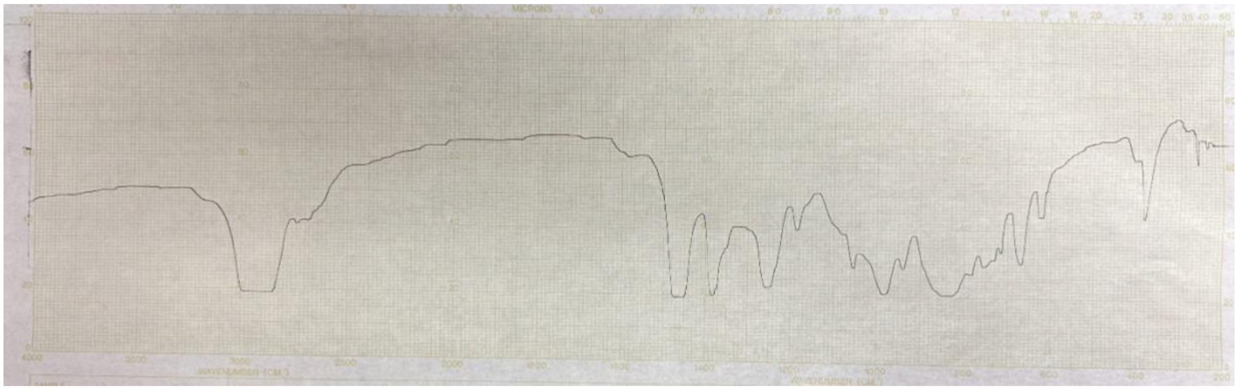

Figure S7. IR spectrum (Nujol) of [K][Ni{N(SiMe$_3$)$_2$}$_2$] (**2**).

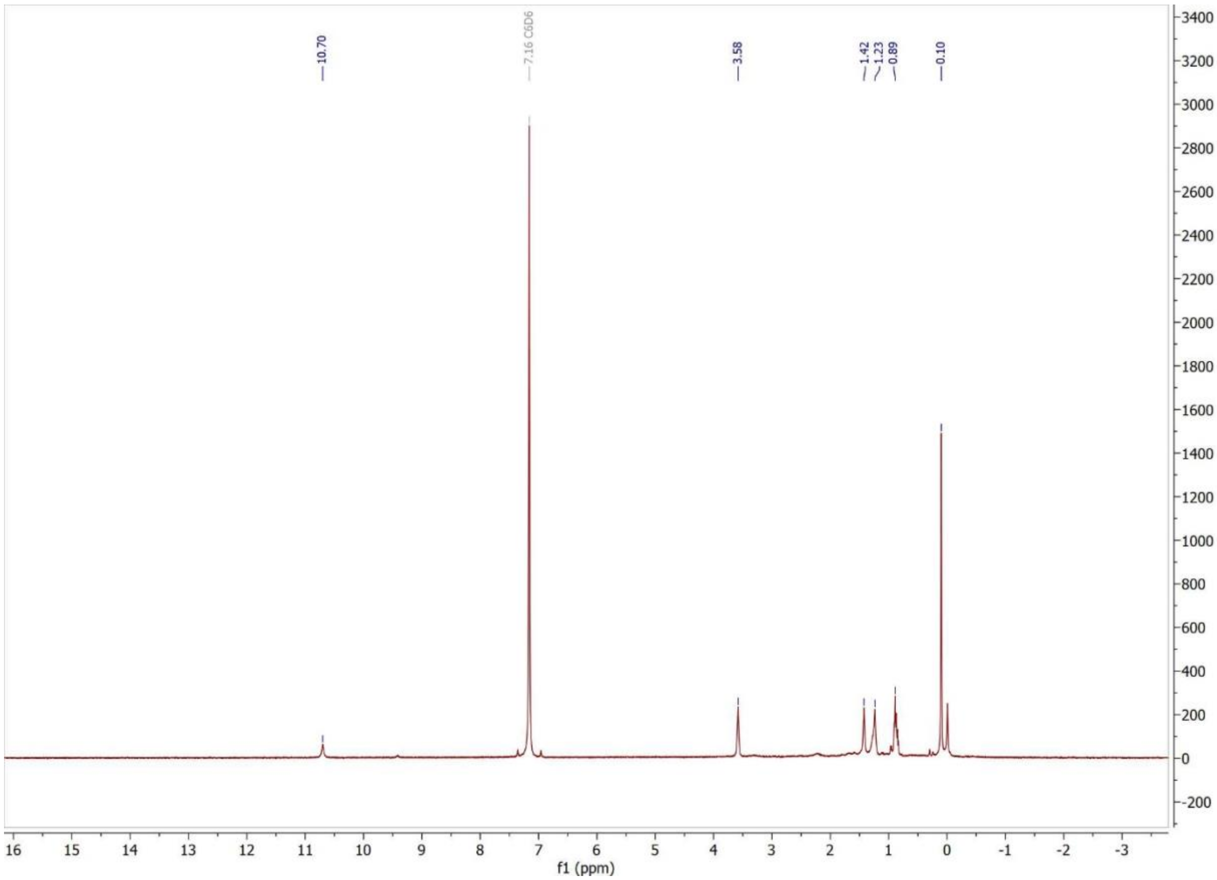

Figure S8. ^{1}H NMR (400 MHz, C_6D_6, 25 ^oC) spectrum of [K(THF)$_2$][Ni(N(SiMe$_3$)$_2$)$_3$] (**3**). Coordinated THF indicated at 3.58 and 1.42 ppm. Residual hexane indicated at 1.23 and 0.89 ppm. Free HMDS indicated at 0.10 ppm.

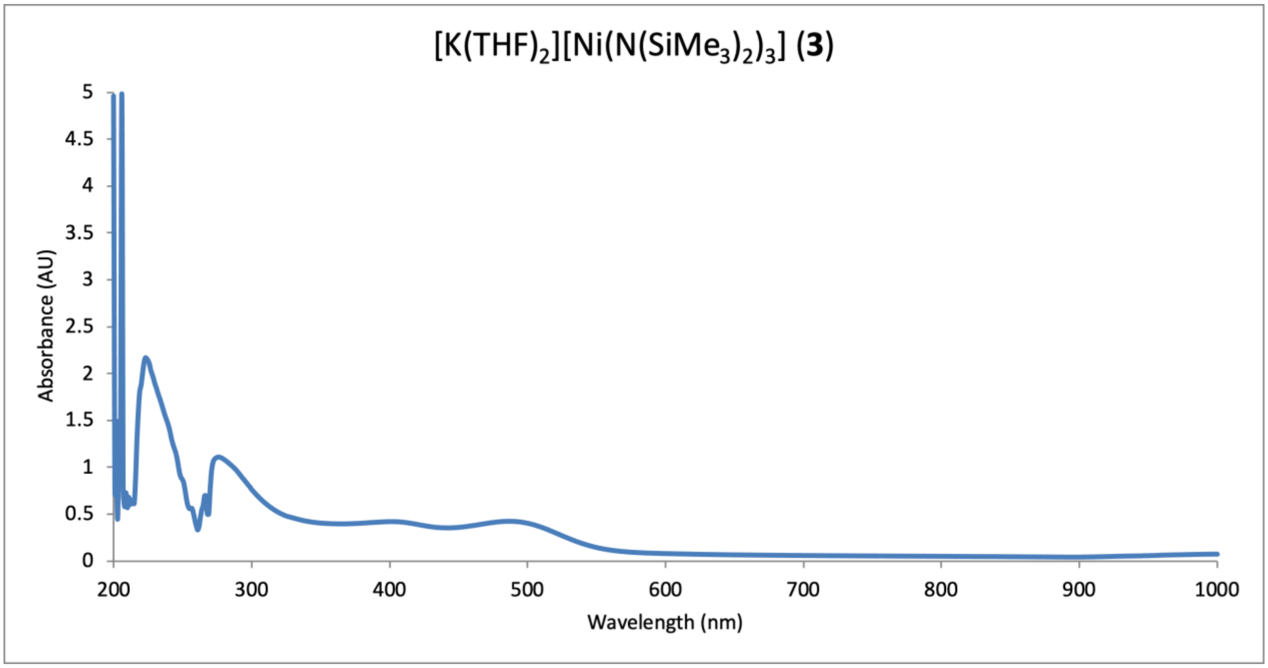

Figure S9. Concentrated (0.645 mM) UV-vis spectrum of $[K(THF)_2][Ni(N(SiMe_3)_2)_3]$ (**3**) in hexane.

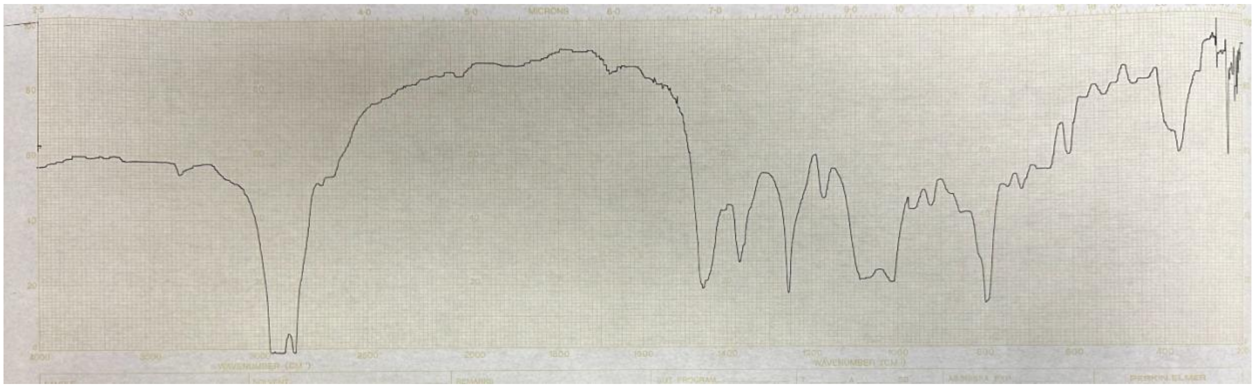

Figure S10. IR spectrum (Nujol)of $[K(THF)_2][Ni(N(SiMe_3)_2)_3]$ (**3**).

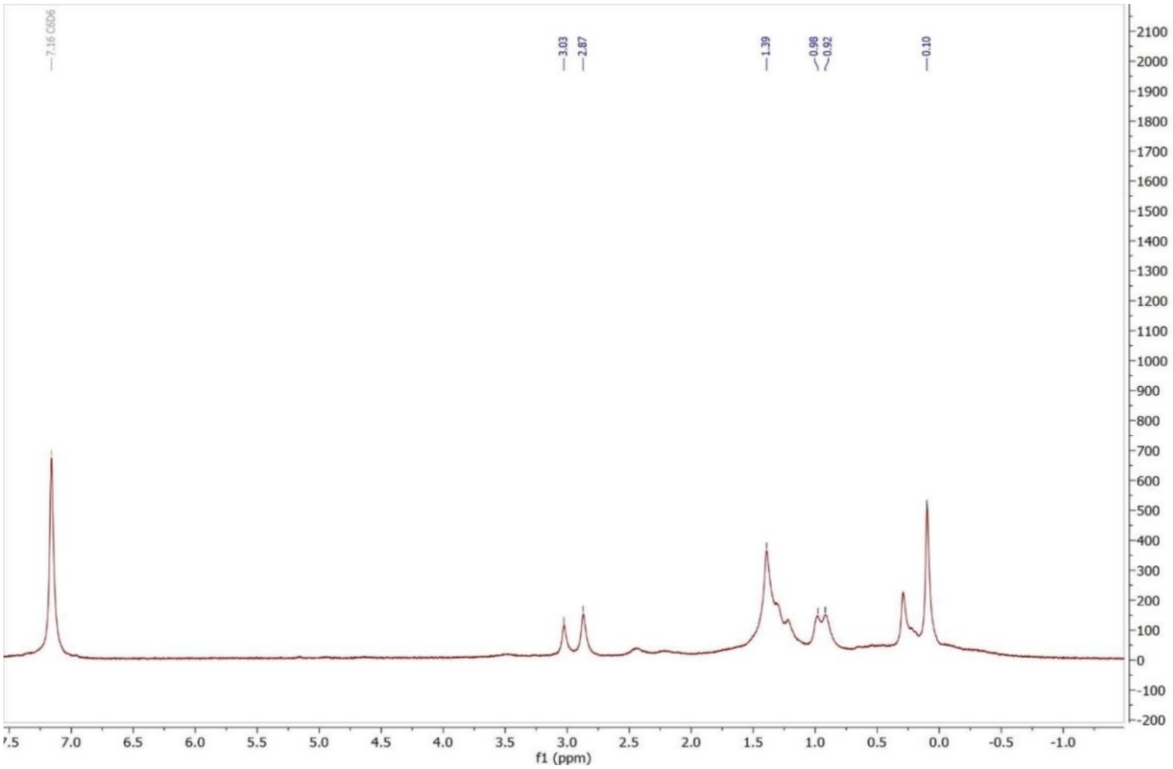

Figure S11. ^{1}H NMR spectrum (400 MHz, C_6D_6, 25 °C) of $[K(DME)][Ni_2(N(SiMe_3)_2)_3]$ (**4**). Coordinated DME indicated at 3.03 and 2.87 ppm. Free HMDS indicated at 0.10 ppm.

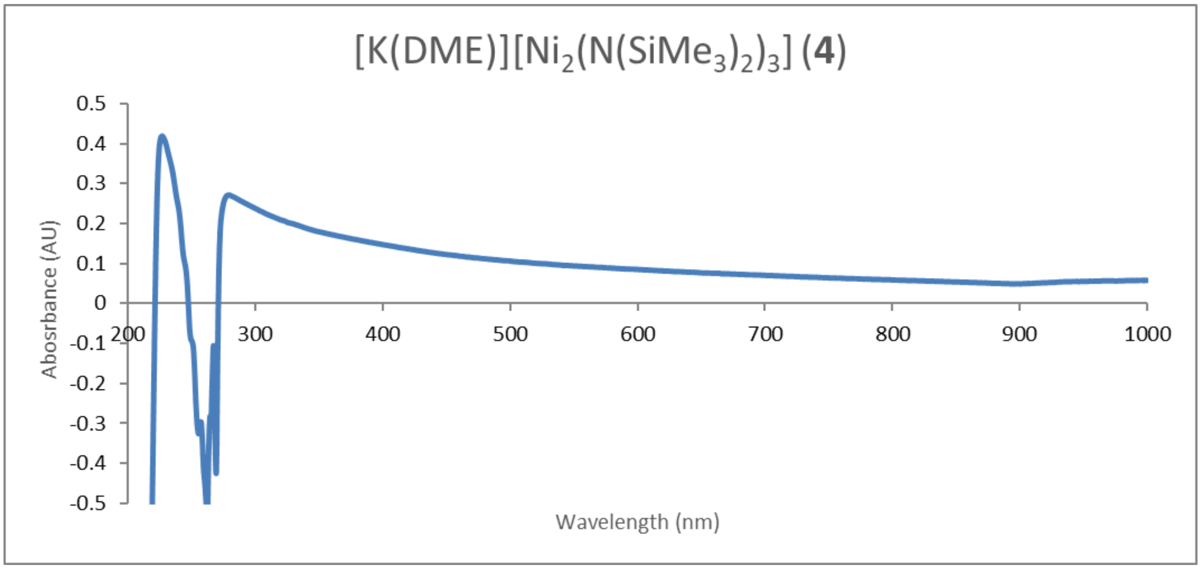

Figure S12. UV-vis spectrum (298 μM) of $[K(DME)][Ni_2(N(SiMe_3)_2)_3]$ (**4**) in hexane.

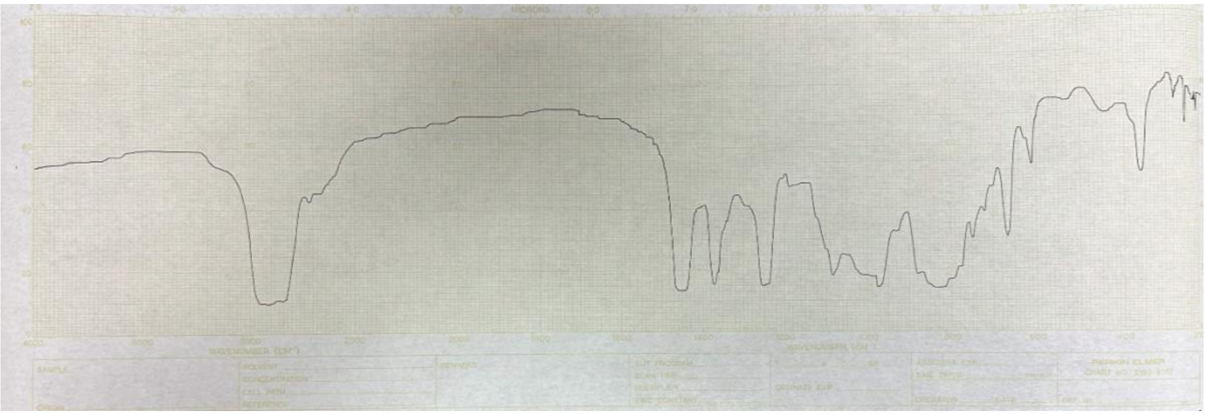

Figure S13. IR spectrum (Nujol) of [K(DME)][$Ni_2(N(SiMe_3)_2)_3$] (**4**).

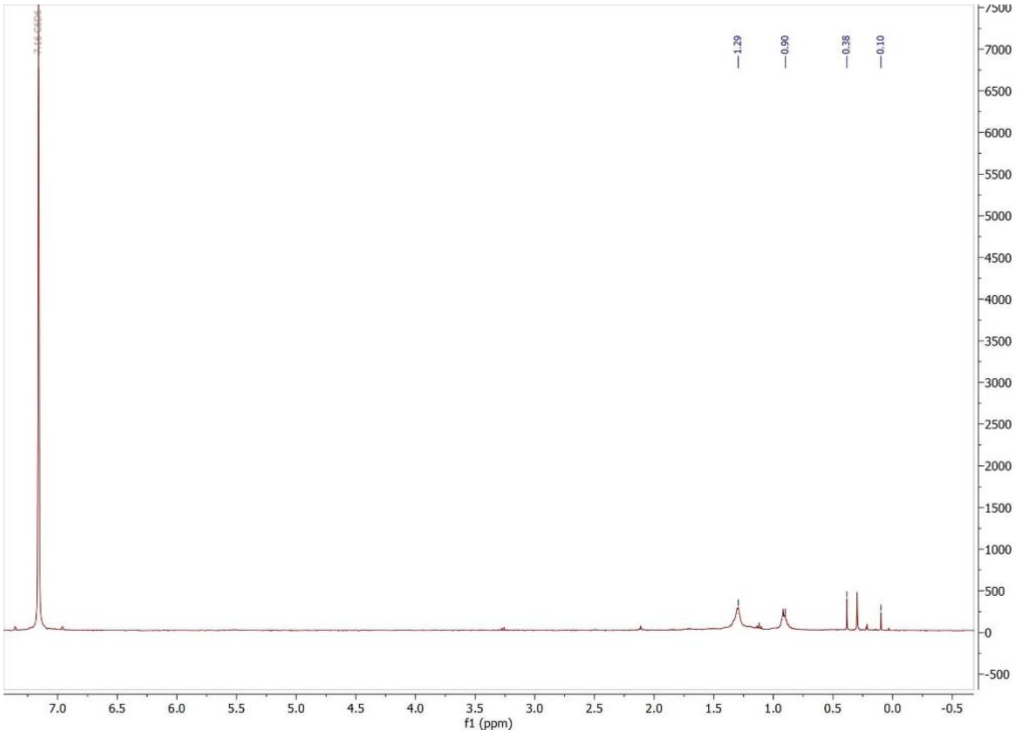

Figure S14. ^{1}H NMR (400 MHz, C_6D_6, 25 ^oC) spectrum of [K_2][O(Ni{$N(SiMe_3)_2$}$_2$)$_2$] (**5**). Residual hexane indicated at 1.29 and 0.90 ppm.

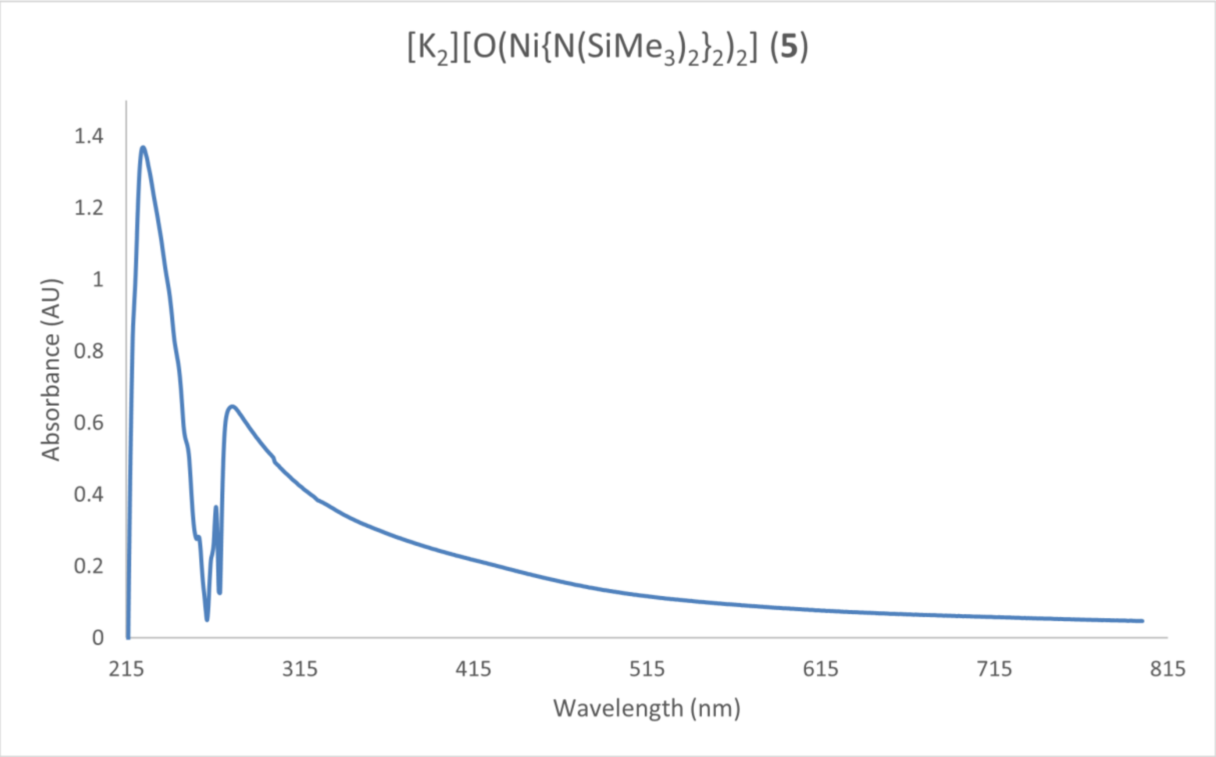

Figure S15. Dilute (234 µM) UV-vis spectrum of $[K_2][O(Ni\{N(SiMe_3)_2\}_2)_2]$ (**5**) in hexane.

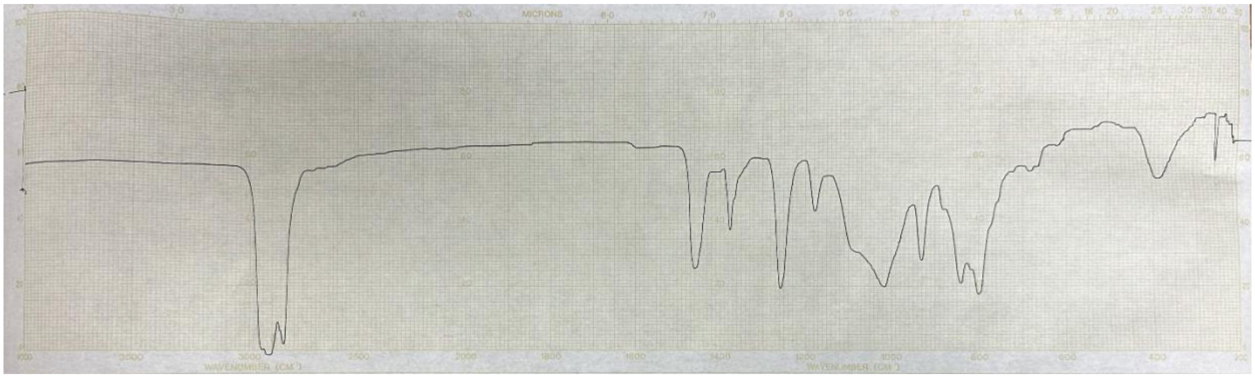

Figure S16. IR spectrum (Nujol) of $[K_2][O(Ni\{N(SiMe_3)_2\}_2)_2]$ (**5**).

Table S1. Crystal data for **1-3**.

Compound Name	$[K][Ni(N(SiMe_3)_2)_3]$ (**1**)	$[K][Ni(N(SiMe_3)_2)_2]$ (**2**)	$[K(THF)_2][Ni(N(SiMe_3)_2)_3]$ (**3**)
Empirical formula	C18 H54 K N3 Ni Si6	C12 H36 K N2 Ni Si4	C26 H70 K N3 Ni O2 Si6
Formula weight	578.99	418.6	723.2
Temperature	190.15 K	190.00 K	90(2) K
Wavelength	0.71073 Å	0.71073 Å	0.71073 Å
Crystal system	Trigonal	Monoclinic	Monoclinic
Space group	R -3 c	P 21/c	C2/c

Unit cell dimensions			
a	a = 27.2362(11) Å	a = 18.5659(6) Å	a = 26.0751(7) Å
b	b = 27.2362(11) Å	b = 6.6370(2) Å	b = 11.3866(3) Å
c	c = 26.4406(17) Å	c = 20.7487(7) Å	c = 18.4807(9) Å
α	α= 90°.	α= 90°.	α= 90°.
β	β= 90°.	β= 114.9330(10)°.	β= 131.3540(10)°.
γ	γ = 120°.	γ = 90°.	γ = 90°.
Volume	16986.1(18) Å3	2318.41(13) Å3	4118.8(3) Å3
Z	18	4	4
Density (calculated)	1.019 Mg/m3	1.199 Mg/m3	1.166 Mg/m3
Absorption coefficient	0.824 mm-1	1.218 mm-1	0.772 mm-1
F(000)	5652	900	1576
Crystal size	0.596 x 0.064 x 0.033 mm3	0.321 x 0.15 x 0.114 mm3	0.208 x 0.207 x 0.057 mm3
Crystal color and habit	yellow Needle	pale blue Rectangular	yellow Rectangular
Diffractometer	Bruker Photon100 CMOS	Bruker Photon2 CMOS	Bruker Photon2 CMOS
Theta range for data collection	2.314 to 27.537°.	1.985 to 30.531°.	2.069 to 27.492°.
Index ranges	-28<=h<=29, -35<=k<=35, -33<=l<=34	-26<=h<=26, -9<=k<=9, -29<=l<=29	-33<=h<=33, -14<=k<=14, -23<=l<=23
Reflections collected	27627	21996	15712
Independent reflections	4355 [R(int) = 0.0455]	7063 [R(int) = 0.0172]	4725 [R(int) = 0.0602]
Observed reflections (I > 2sigma(I))	3343	6171	3507
Completeness to theta = 25.242°	99.90%	99.70%	99.80%
Absorption correction	Numerical, SADABS (multi-scan)	Empirical, SADABS (multi-scan)	Empirical, SADABS (multi-scan)
Max. and min. transmission	0.9560 and 0.5573	0.7385 and 0.8374	0.9010 and 0.7581
Solution method	SHELXT 2014/5 (Sheldrick, 2014)	SHELXT 2014/5 (Sheldrick, 2014)	SHELXT 2014/5 (Sheldrick, 2014)
Refinement method	XL (Sheldrick, 2008) Full-matrix least-squares on F2	XL (Sheldrick, 2008) Full-matrix least-squares on F2	SHELXL-2016/6 (Sheldrick, 2016) Full-matrix least-squares on F2
Data / restraints / parameters	4355 / 0 / 142	7063 / 0 / 193	4725 / 21 / 236
Goodness-of-fit on F2	1.083	1.046	1.011
Final R indices [I>2sigma(I)]	R1 = 0.0343, wR2 = 0.0865	R1 = 0.0240, wR2 = 0.0635	R1 = 0.0352, wR2 = 0.0736
R indices (all data)	R1 = 0.0503, wR2 = 0.0924	R1 = 0.0289, wR2 = 0.0663	R1 = 0.0591, wR2 = 0.0823
Extinction coefficient	n/a	n/a	n/a
Largest diff. peak and hole	0.452 and -0.504 e.Å-3	0.558 and -0.572 e.Å-3	0.440 and -0.330 e.Å-3

Table S2. Crystal data for **4-5**.

Compound Name	[K(DME)][$Ni_2(N(SiMe_3)_2)_3$] (**4**)	[K_2][O(Ni{$N(SiMe_3)_2$}$_2$)$_2$] (**5**)
Empirical formula	C22 H64 K N3 Ni2 O2 Si6	C24 H72 K2 N4 Ni2 O Si8
Formula weight	727.82	853.19
Temperature	190.00 K	100(2) K
Wavelength	1.54178 Å	0.71073 Å
Crystal system	Orthorhombic	Triclinic
Space group	Pbca	P-1
Unit cell dimensions		
a	a = 21.1303(3) Å	a = 9.0243(6) Å
b	b = 17.4248(2) Å	b = 10.9286(8) Å
c	c = 22.3814(3) Å	c = 12.8609(9) Å
α	α= 90°.	α= 97.717(2)°.
β	β= 90°.	β= 109.022(2)°.
γ	γ = 90°.	γ = 101.511(2)°.
Volume	8240.64(19) Å3	1147.48(14) Å3
Z	8	1
Density (calculated)	1.173 Mg/m3	1.235 Mg/m3
Absorption coefficient	3.872 mm-1	1.233 mm-1
F(000)	3136	458
Crystal size	0.33 x 0.193 x 0.078 mm3	0.268 x 0.175 x 0.054 mm3
Crystal color and habit	red Rectangular	colourless Rectangular
Diffractometer	Bruker Photon2 CMOS	Bruker Photon2 CMOS
Theta range for data collection	3.836 to 72.171°.	2.302 to 28.288°.
Index ranges	-25<=h<=25, -21<=k<=19, -27<=l<=27	-12<=h<=12, -14<=k<=14, -17<=l<=17
Reflections collected	36520	11267
Independent reflections	8066 [R(int) = 0.0293]	5684 [R(int) = 0.0125]
Observed reflections (I > 2sigma(I))	6949	5183
Completeness to theta = 25.242°	99.60%	99.90%
Absorption correction	Empirical, SADABS (multi-scan)	Empirical, SADABS (multi-scan)
Max. and min. transmission	0.5933 and 0.4663	0.8621 and 0.7565
Solution method	SHELXT 2014/5 (Sheldrick, 2014)	SHELXT 2014/5 (Sheldrick, 2014)
Refinement method	XL (Sheldrick, 2008) Full-matrix least-squares on F2	SHELXL-2016/6 (Sheldrick, 2016) Full-matrix least-squares on F2
Data / restraints / parameters	8066 / 359 / 642	5684 / 0 / 199
Goodness-of-fit on F2	1.105	1.061
Final R indices [I>2sigma(I)]	R1 = 0.0358, wR2 = 0.1061	R1 = 0.0216, wR2 = 0.0560
R indices (all data)	R1 = 0.0403, wR2 = 0.1096	R1 = 0.0250, wR2 = 0.0581

Extinction coefficient	n/a	n/a
Largest diff. peak and hole	0.307 and -0.297 e.Å-3	0.607 and -0.242 e.Å-3

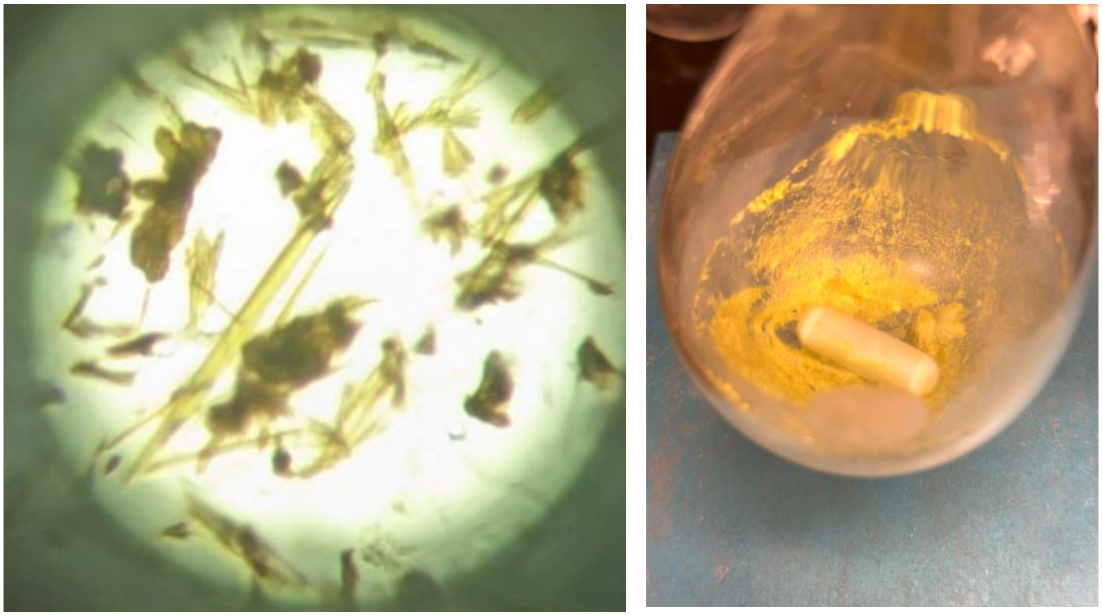

Figure S17. Crystalline [K][Ni(N(SiMe$_3$)$_2$)$_3$] (**1**).

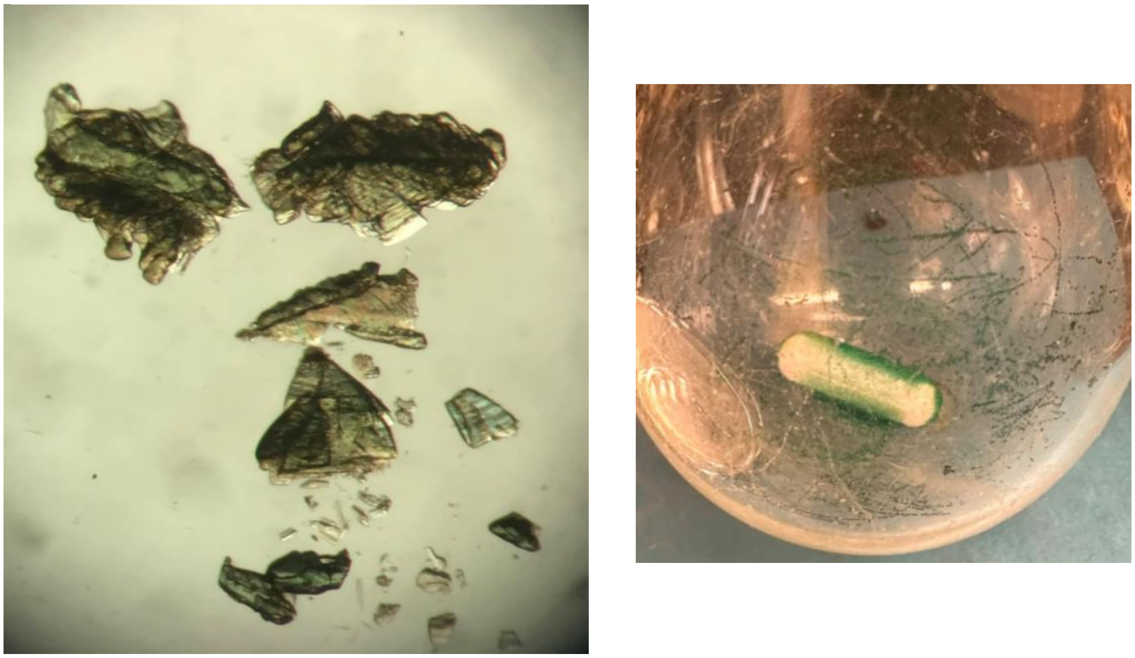

Figure S18. Crystalline [K][Ni(N(SiMe$_3$)$_2$)$_2$] (**2**).

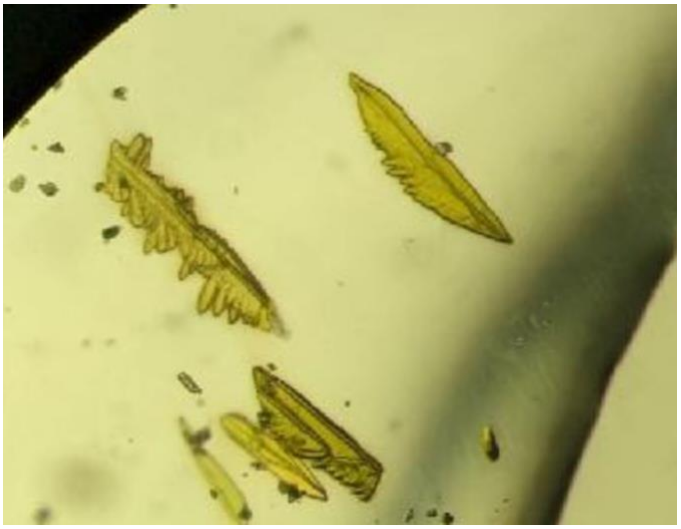

Figure S19. Crystalline $[K(THF)_2][Ni\{N(SiMe_3)_2\}_3]$ (**3**).

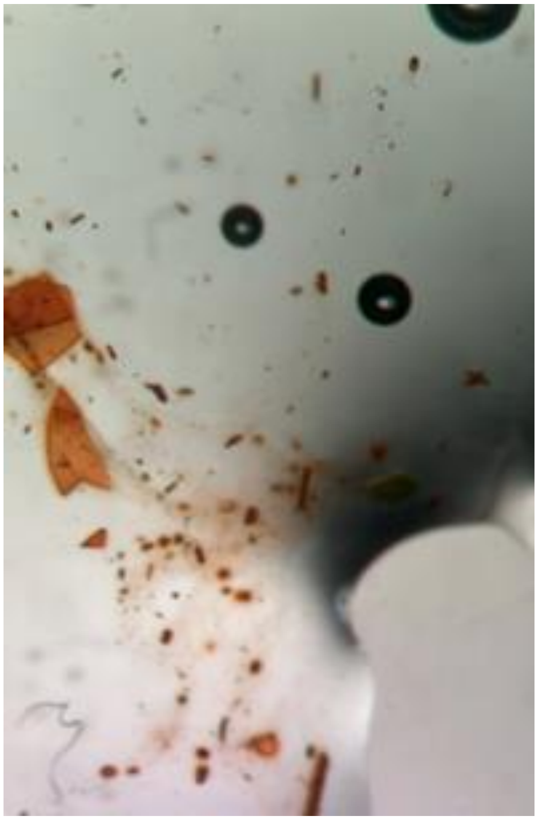

Figure S20. Crystalline $[K(DME)][Ni_2\{N(SiMe_3)_2\}_3]$ (**4**).

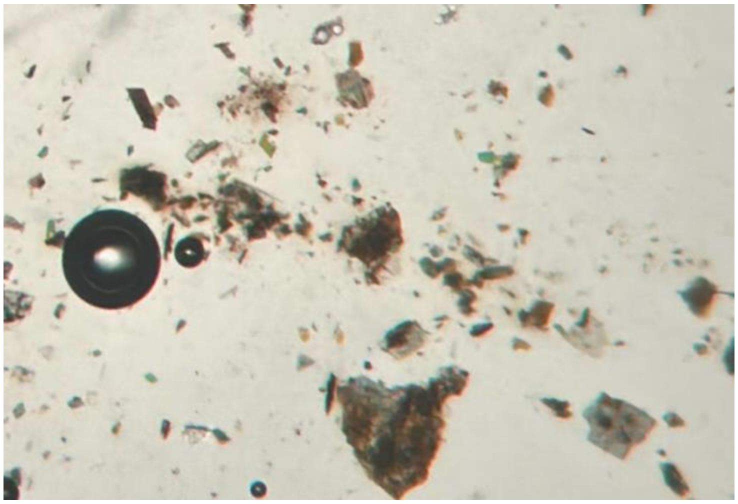

Figure S21. Colorless, crystalline $[K_2][O(Ni\{N(SiMe_3)_2\}_2)_2]$ (**5**) under a polarization filter.

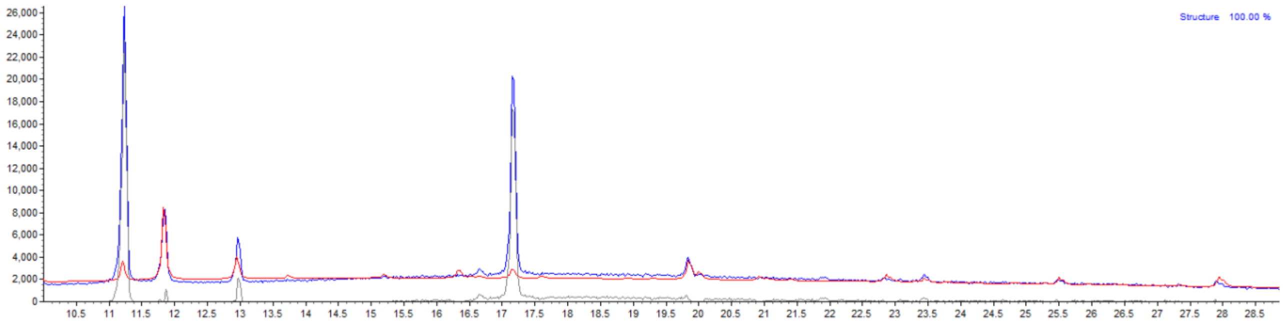

Figure S22. PXRD pattern of **1**. The experimentally obtained pattern is shown in blue and the calculated pattern is shown in red. Data processed in TOPAS (Bruker) v.5.

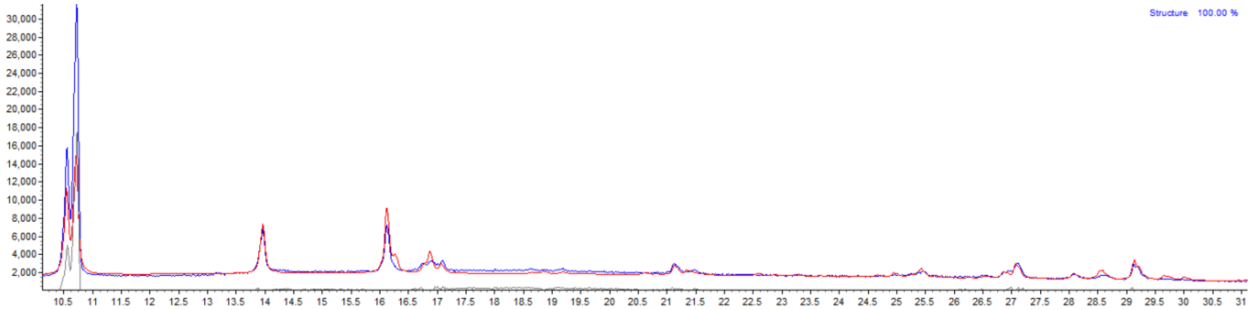

Figure S23. PXRD pattern of **2**. The experimentally obtained pattern is shown in blue and the calculated pattern is shown in red. Data processed in TOPAS (Bruker) v.5.
